# Measurable Residual Disease Detected by Multiparameter Flow Cytometry and Sequencing Improves Prediction of Relapse and Survival in Acute Myeloid Leukemia

**DOI:** 10.3389/fonc.2021.677833

**Published:** 2021-05-20

**Authors:** Fu-Jia Liu, Wen-Yan Cheng, Xiao-Jing Lin, Shi-Yang Wang, Tian-Yi Jiang, Ting-Ting Ma, Yong-Mei Zhu, Yang Shen

**Affiliations:** Shanghai Institute of Hematology, State Key Laboratory of Medical Genomics, National Research Centre for Translational Medicine at Shanghai, Ruijin Hospital Affiliated to Shanghai Jiao Tong University School of Medicine, Shanghai, China

**Keywords:** acute myeloid leukemia, measurable residual disease, molecular markers, multiparameter flow cytometry, prognosis

## Abstract

The clinically ideal time point and optimal approach for the assessment of measurable residual disease (MRD) in patients with acute myeloid leukemia (AML) are still inconclusive. We investigated the clinical value of multiparameter flow cytometry-based MRD (MFC MRD) after induction (n = 492) and two cycles of consolidation (n = 421). The latter time point was proved as a superior indicator with independent prognostic significance for both relapse-free survival (RFS, HR = 3.635, 95% CI: 2.433–5.431, P <0.001) and overall survival (OS: HR = 3.511, 95% CI: 2.191–5.626, P <0.001). Furthermore, several representative molecular MRD markers were compared with the MFC MRD. Both approaches can establish prognostic value in patients with *NPM1* mutations, and *FLT3*, *C-KIT*, or *N-RAS* mutations involved in kinase-related signaling pathways, while the combination of both techniques further refined the risk stratification. The detection of *RUNX1–RUNX1T1* fusion transcripts achieved a considerable net reclassification improvement in predicting the prognosis. Conversely, for patients with biallelic *CEBPA* or *DNMT3A* mutations, only the MFC method was recommended due to the poor prognostic discriminability in tracking mutant transcripts. In conclusion, this study demonstrated that the MFC MRD after two consolidation cycles independently predicted clinical outcomes, and the integration of MFC and molecular MRD should depend on different types of AML-related genetic lesions.

## Introduction

Acute myeloid leukemia (AML) is a group of hematological malignant disorders characterized by the high heterogeneity in clinical manifestation, genetic abnormalities, and prognosis (1). Via the treatment modalities as exemplified by cytotoxic drugs or molecular targeted therapies, a high complete remission (CR) rate could be achieved, however, a substantial proportion of AML patients will relapse due to residual leukemic cells below the detection threshold of traditional morphologic methods. To achieve a long term remission and survival, post-remission therapies should be administered not only based on pre-treatment parameters including age, cytogenetics, and a limited set of molecular genetic markers, but also on several post-treatment factors, among which the detection of residual disease is of utmost importance (2, 3).

Measurable residual disease (MRD; also named minimal residual disease) detected in AML patients with hematological complete remission after treatment has been suggested as a powerful prognostic indicator (4, 5). In general clinical practice, MRD is reliably monitored using the two most common methods, multiparameter flow cytometry-based MRD (MFC MRD) and polymerase chain reaction (PCR)-based MRD (Gene MRD), quantifying MRD in AML by virtue of either immunophenotype or molecular abnormalities of leukemic cells. However, until now, issues on when and how to apply the evaluation of MRD in daily clinical practice are still controversial.

Numerous studies have highlighted the prognostic impact of MFC MRD assessment at diverse time points in AML patients, as exemplified by post-induction (PI), post-consolidation (PC), before and after hematopoietic stem cell transplantation (HSCT) (6–9), whereas the clinically ideal time point for MRD assessment is inconclusive. On the other side, the existing two common approaches differ in sensitivity and applicability. The MFC MRD can be monitored in the majority of AML patients with rapid turnaround time, but an established threshold with considerably high sensitivity and reproducibility is still an unmet clinical need (7, 10). Conversely, although the PCR technique is highly sensitive, its application is limited to a fraction of AML patients who harbored specific genetic aberrations suitable for MRD detection, including *RUNX1–RUNX1T1*, *CBFB–MYH11*, and *PML–RARα* fusions, and *NPM1* mutations (5, 11–16). Notably, the next-generation sequencing (NGS) has introduced novel molecular markers for MRD assessment, and reliable criteria for their routine application in the clinical setting are under exploration. Consequently, how to compare and integrate current procedures for MRD monitoring is of great clinical significance.

In this study, we evaluated the prognostic value of the MFC MRD after induction and two consolidation cycles in AML to identify the optimal time point for MRD measurement. Furthermore, the tracking of molecular MRD on a series of AML-related gene alterations was compared with the MFC MRD, highlighting the necessity of combining both MFC and molecular techniques to establish an integrated methodology for MRD monitoring.

## Materials and Methods

### Patients and Treatment

From January 2011 to June 2018, a total of 833 consecutive newly diagnosed AML (non-M3) patients treated in Ruijin Hospital were enrolled. Among which, the majority of patients diagnosed after 2015 participated in one of three phase II/III clinical trials, which were registered at the Chinese Clinical Trial Registry (www.chictr.org.cn: ChiCTR-OPC-15006085; ChiCTR-OIC-16007764; ChiCTR-OIN-16008955).

Young patients (<60 years) were given standard intensive “3 + 7” IA/DA-based regimens as initial induction, which contained idarubicin/daunorubicin (10–12 mg/m^2^/45–60 mg/m^2^, D1–3) and cytarabine (100–200 mg/m^2^ D1–7). When CR was achieved, four cycles of high-dose cytarabine (2 g/m^2^) were delivered as consolidation. Elderly patients (≥60 years) were evaluated for the fitness by the treating physician. Fit patients received the same induction regimens as young patients but reduced the consolidation to two cycles of high-dose cytarabine. Unfit patients received either low dose “3 + 7” regimens, demethylation agents, or other less intensive therapies at the discretion of the physician. Treatment protocols of the three clinical trials are provided in **Supplementary Methods**.

### Multiparameter Flow Cytometry

Bone marrow aspirate samples were obtained at diagnosis and before the first and third cycles of consolidation chemotherapy, which were processed through the standard procedure of our institution (17). The MFC MRD was monitored by using the 10-color flow cytometry, and monoclonal antibodies against 21 antigens, including stem cell and progenitor markers (CD34, CD38, CD45, CD117, CD123, and HLA-DR), myelomonocytic markers (CD13, CD11b, CD14, CD15, CD33, MPO, and CD64), and lymphoid lineage markers (CD2, cyCD3, CD4, CD7, CD19, cyCD79a, TdT, and CD56) were utilized. Identical antibody-fluorochrome combinations at diagnosis and during the follow-up period were utilized for tracking established LAIPs and newly emerging aberrant immunophenotypes. For statistical analyses, the “LAIP‐based different‐from‐normal approach” was applied. Detailed definitions concerning MFC are provided in **Supplementary Methods**.

### Molecular Events

Molecular alterations of AML in this study were selected according to the established laboratory developed tests (LDTs) at Shanghai Institute of Hematology based on our previous work conducted in a large AML cohort, in which gene mutations and fusions showing significant prognostic value were then tested in daily clinical routine (18). Genetic alterations including *FLT3-*ITD/TKD, *KMT2A-*PTD, *NPM1*, *NRAS*, *CKIT*, *CEBPA*, *DNMT3A*, *IDH1*, and *IDH2* were detected by PCR and Sanger sequencing. *RUNX1–RUNX1T1*, *CBFβ–MYH11*, and *KMT2A*-related fusion genes were detected *via* reverse transcription (RT)-PCR strategy as previously reported (19). The level of *RUNX1–RUNX1T1* transcripts was measured by quantitative real-time PCR (qPCR), and a >3-log reduction compared with the baseline level at diagnosis was defined as molecular MRD negativity according to the published literature (14, 20).

### Statistical Analyses

Kaplan–Meier and hazard ratio analyses were used to calculate and compare the relapse-free survival (RFS) and overall survival (OS). The Cox proportional hazard regression model was applied for the multivariate analysis of independent factors for RFS and OS. To investigate the prognostic accuracy of MRD status by the MFC and molecular methods, the net reclassification improvement (NRI) (21) was used to measure the net gain in risk reclassification between different techniques for MRD monitoring. All of the statistical procedures mentioned above were carried out using the R (version 4.0.0) and the SPSS (version 26.0) software packages.

## Results

### Characteristics of Patients and Their Associations With MFC MRD Status

**Figure 1:**
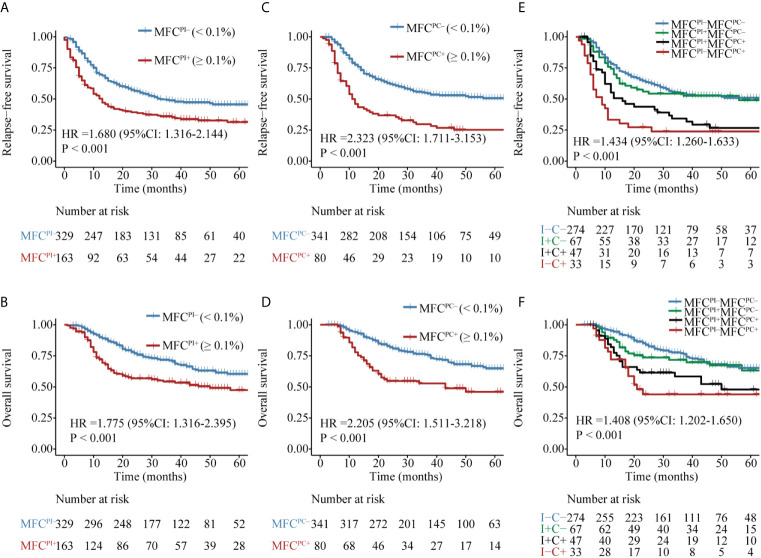
Kaplan–Meier curves for probability of relapse-free survival (RFS) and overall survival (OS) in AML patients. **(A, B)** RFS and OS based on MFC MRD status post induction. **(C, D)** RFS and OS based on MFC MRD status post two consolidation cycles. **(E, F)** RFS and OS based on MFC MRD status in the combination of both time points.

The patient flow diagram is depicted in **Figure S1**. Among the 833 AML patients, 639 (76.7%) achieved CR after induction chemotherapy, of whom 587 (91.9%) patients had specific LAIPs at diagnosis that were suitable for MRD monitoring by flow cytometry. The genetic alterations of AML patients stratified by LAIPs are shown in **Table S1**, and the most frequent LAIPs are summarized in **Table S2**. A higher frequency of *RUNX1–RUNX1TI* (P = 0.031) and biallelic *CEBPA* (Bi*CEBPA*, P = 0.001) mutations, but lower frequency of *CBFβ–MYH11* (P = 0.009) were observed in patients who had LAIPs at diagnosis. MRD analysis by MFC was available in 492 patients after induction, among which, 24 patients chose HSCT as consolidation, 41 patients relapsed, and six patients died. Consequently, 421 patients who were treated with chemotherapy only remained in CR and received MRD monitoring after two cycles of consolidation therapy. By comparing different cut-off levels including 0, 0.01, 0.035, 0.1, and 1% to distinguish MRD^+^ from MRD^-^ patients, the cut-off of 0.1% was proved to be most relevant to prognosis, displaying significant disparities in both RFS and OS (**Figure 1**, **Figure S2**). Therefore, the level of ≥0.1% was considered as MRD positive in this study. As shown in **Table 1**, when one to two cycles of induction chemotherapy were completed, 329 (66.9%) of the 492 patients were classified into MFC MRD negative group, termed as MFC^PI–^. Moreover, 340 (81%) of 421 achieved MFC MRD negativity after two cycles of consolidation chemotherapy, termed as MFC^PC–^. There were no significant differences in age, gender, peripheral blood count, and BM blasts between MFC^–^ and MFC^+^ at both time points. Patients in the MFC^PI+^ group were less likely to carry *RUNX1–RUNX1T1* (P = 0.003), and those with MFC^PC+^ were more likely to harbor biallelic *CEBPA* (Bi*CEBPA*) mutations at diagnosis (P = 0.003). In cytogenetic risk stratification, favorable cytogenetic abnormalities were less common in patients with MFC^PI+^ (P = 0.002), while a higher frequency of unfavorable risk was seen in the MFC^PI+^ group (P = 0.003). Besides, patients who required two induction cycles to attain CR were more likely to have MFC^PI+^ (P = 0.013). The follow-up of all patients ended in April 2020, with a median follow-up time of 45 (range 1–108) months.

**Table 1:** Clinical characteristics of AML patients.

	All patients(n = 492)	MFC MRD PI (n = 492)			MFC MRD PC (n = 421)		
Clinical characteristics		MFC^PI−^(n = 329)	MFC^PI+^(n = 163)	P	MFC^PC−^(n = 341)	MFC^PC+^(n = 80)	P
**Age (y)**							
Median	45	45	44	0.635	44	44	0.354
Range	15–74	15–73	15–74		15–71	16–74	
<60	412 (83.7)	276 (83.9)	136 (83.4)	1.000	295 (86.5)	64 (80.0)	0.354
≥60	80 (16.3)	53 (16.1)	27 (16.6)		46 (13.5)	16 (20.0)	
**Gender, n (%)**				0.436			0.124
Male	270 (54.9)	176 (53.5)	94 (57.7)		178 (52.2)	50 (62.5)	
Female	222 (45.1)	153 (46.5)	69 (42.3)		163 (47.8)	30 (37.5)	
**WBC count, ×10^9^/L**				0.749			0.929
Median	11.4	11.9	9.76		11.4	11.08	
Range	0.5–291	0.5–291	0.9–239		0.5–291	0.8–213	
**HB, g/L**				0.521			0.274
Median	89.5	87.5	91		89	96	
Range	3–171	30–164	3–171		3–171	37–137	
**PLT count, ×10^9^/L**				0.885			0.476
Median	44.5	45	44		46	35	
Range	3–490	3–490	5–275		4–490	5–329	
**BM blasts, %**				0.536			0.445
Median	64	65	64		65	61.5	
Range	6–98	7–98	6–98		7–98	6–96	
**WHO category, n%**							
**AML with recurrent genetic abnormalities**							
AML with t(8;21); *RUNX1–RUNX1T1*	74 (15.0)	61 (18.5)	13 (8.0)	0.003	61 (17.9)	8 (10.0)	0.122
AML with inv(16) or t(16;16); *CBFB-MYH11*	30 (6.1)	24 (7.3)	6 (3.7)	0.169	25 (7.3)	5 (6.2)	0.923
AML with t(9;11); *MLLT3-KMT2A*	9 (1.8)	7 (2.1)	2 (1.2)	0.731	6 (1.8)	0 (0.0)	0.502
Provisional entity: AML with *BCR-ABL1*	1 (0.2)	1 (0.3)	0 (0.0)	1.000	1 (0.3)	0 (0.0)	1.000
AML with mutated *NPM1*	100 (20.3)	72 (21.9)	28 (17.2)	0.270	75 (22.0)	12 (15.0)	0.216
AML with biallelic mutations of *CEBPA*	97 (19.7)	64 (19.5)	33 (20.2)	0.930	64 (18.8)	28 (35.0)	0.003
**AML, NOS**							
AML without maturation	2 (0.4)	2 (0.6)	0 (0.0)	0.807	2 (0.6)	0 (0.0)	1.000
AML with maturation	12 (2.4)	9 (2.7)	3 (1.8)	0.768	9 (2.6)	2 (2.5)	1.000
Acute myelomonocytic leukemia	50 (10.2)	30 (9.1)	20 (12.3)	0.352	33 (9.7)	5 (6.2)	0.456
Acute monoblastic/monocytic leukemia	60 (12.2)	26 (7.9)	34 (20.9)	<0.001	28 (8.2)	11 (13.8)	0.186
Pure erythroid leukemia	12 (2.4)	10 (3.0)	2 (1.2)	0.360	10 (2.9)	1 (1.2)	0.646
Not classified	45 (9.1)	23 (7.0)	22 (13.5)	0.029	27 (7.9)	8 (10.0)	0.702
**2017 ELN cytogenetic stratification**							
Favorable	118 (25.4)	94 (29.8)	24 (15.9)	0.002	97 (29.7)	14 (19.2)	0.096
Intermediate	287 (61.7)	190 (60.5)	97 (64.2)	0.501	197 (60.2)	48 (65.8)	0.459
Unfavorable	60 (12.9)	30 (9.6)	30 (19.9)	0.003	33 (10.1)	11 (15.1)	0.307
**Number of induction cycles to attain CR, n (%)**				0.013			0.339
one cycle	429 (87.2)	296 (90.0)	133 (81.6)		309 (90.6)	69 (86.2)	
two cycles	63 (12.8)	33 (10.0)	30 (18.4)		32 (9.4)	11 (13.8)	
**HSCT, n (%)**	145 (29.5)	89 (27.1)	56 (34.4)	0.117	89 (26.1)	28 (35.0)	0.144

WBC, white blood cell; HB, hemoglobin; PLT, platelet; BM, bone marrow; WHO, The World Health Organization; NOS, not otherwise specified; MFC, multicolor flow cytometry; MRD, measurable residual disease; PI, post induction; PC, post the second consolidation; MFC^PI−^, MFC MRD negative post induction; MFC^PI+^, MFC MRD positive post induction; MFC^PC−^, MFC MRD negative post the second consolidation; MFC^PC+^, MFC MRD positive post the second consolidation; HSCT, hematopoietic stem cell transplantation.

### Prognostic Significance of MFC MRD at Different Time Points

Patients with MFC^PI–^ (median MRD, 0; range, 0–0.09%) had a significantly favorable RFS and OS than those with MFC^PI+^ (median MRD, 0.43%; range, 0.1–11.4%) (**Figures 1A, B** and **Table S3**). Similarly, patients in the MFC^PC–^ group (median MRD, 0; range, 0–0.09%) had a better prognosis than those whose MFC MRD status was positive (median MRD, 0.27%; range, 0.1–4.81%) (**Figures 1C, D** and **Table S3**). Of note, the status of MFC^PC^ seemed to provide a better discrimination ability for both short- and long-term survival than that of MFC^PI^.

The dynamics of MFC MRD status after induction and the second cycle of consolidation therapy in the 421 patients who experienced MFC MRD monitoring at both time points were integratively evaluated. Patients were stratified into four groups based on the MFC MRD status at the two checkpoints. As shown in **Figures 1E, F**, there was no difference in the distribution of RFS and OS between MFC^PI–^MFC^PC–^ and MFC^PI+^MFC^PC–^ patients (P = 0.787), and between patients with MFC^PI+^MFC^PC+^ and MFC^PI-^MFC^PC+^ (P = 0.408), while both groups conferred an inferior prognosis compared to those with MFC^PC–^. Intriguingly, the prognostic impact of the MFC^PC^ MRD status in both MFC^PI–^ and MFC^PI+^ patients was significant (**Figures 1E, F** and **Table S3**). The prognostic value of the MFC^PC^ MRD status was also observed in young and old AML patients, respectively (**Figures S3, S4**), and in the ELN low and intermediate cytogenetic risk group, respectively (**Figures S5, S6**), while it was not significant in the high-risk group (**Figure S7**).

### The Post-Consolidation MFC MRD Was an Independent Prognostic Factor

Age, gender, WBC count, hemoglobin (Hb), platelet count, BM blasts, cytogenetic risk stratification, recurrent genetic alterations at diagnosis, number of induction cycles to CR, HSCT, MFC^PI^, and MFC^PC^ MRD status were included in the univariate analysis for both RFS and OS (**Table S4**), in which factors with P <0.1 were entered into the multivariable Cox analysis. As shown in **Table 2**, after adjusting the impact of well-established prognostic indicators such as age, cytogenetic risk stratification, *FLT3*-ITD, *CKIT*, and Bi*CEBPA* mutations at diagnosis, the post-consolidation MFC MRD status was independently associated with both RFS and OS of AML patients (RFS: HR = 3.635, 95% CI: 2.433–5.431, P <0.001; OS: HR = 3.511, 95% CI: 2.191–5.626, P <0.001). In the ELN intermediate cytogenetic risk group, the MFC^PC^ MRD was also an independent prognostic factor for both RFS and OS (**Tables S5, S6**).

**Table 2:** Multivariate Analysis of Prognostic Variables for RFS and OS.

Variables	RFS		OS		
	HR (95% CI)	P	HR (95% CI)	P
Age	1.018 (1.006–1.030)	0.004	1.021 (1.006–1.037)	0.008
WBC at diagnosis(×10^9^/L) (>100 *vs* ≤100)	1.243 (0.588–2.624)	0.569	1.944 (0.848–4.456)	0.116
HB at diagnosis	1.000 (0.994–1.007)	0.912	0.999 (0.990–1.007)	0.733
BM Blasts	1.004 (0.996–1.012)	0.323	1.008 (0.998–1.018)	0.112
2017ELN risk classification				
Intermediate *vs* Favorable	1.122 (0.727–1.732)	0.604	1.252 (0.718–2.186)	0.428
Unfavorable *vs* Favorable	1.247 (0.936–1.662)	0.132	1.184 (0.822–1.704)	0.365
*FLT3*-ITD	2.137 (1.251–3.652)	0.005	3.175 (1.741–5.789)	<0.001
*CKIT*	1.882 (1.134–3.125)	0.015	2.490 (1.317–4.706)	0.005
Bi*CEBPA*	0.408 (0.252–0.660)	<0.001	0.343 (0.182–0.646)	0.001
CR achieved: 2 cycles vs 1 cycle	0.990 (0.700–1.401)	0.956	0.650 (0.408–1.038)	0.071
HSCT*	0.922 (0.580–1.464)	0.729	0.823 (0.462–1.468)	0.510
MFC^PI+^ *vs* MFC^PI−^	1.050 (0.715–1.543)	0.804	1.303 (0.810–2.096)	0.275
MFC^PC+^ *vs* MFC^PC−^	3.635 (2.433–5.431)	<0.001	3.511 (2.191–5.626)	<0.001

*patients accepted HSCT after the second consolidation were included in multivariate models for RFS and OS.

RFS, relapse-free survival; OS, overall survival. HR, Hazard ratio; CI, confidence interval; WBC, white blood cell; HB, hemoglobin; BM, bone marrow; CR, complete remission; HSCT, hematopoietic stem cell transplantation; MRD, measurable residual disease; MFC^PI+^, MFC MRD positive post induction; MFC^PI−^, MFC MRD negative post induction; MFC^PC+^, MFC MRD positive two cycles of consolidation; MFC^PC−^, MFC MRD negative post two cycles of consolidation.

### Comparison of MRD Assessment by Different Detection Modalities

The clinical utility of molecular MRD and MFC MRD in diverse types of genetic abnormalities was compared in patients with MRD monitoring by both methods after two consolidation cycles. A series of gene markers either of fusions or mutations with certain incidences were selected and described as follows according to gene type and function.

**Figure 2:**
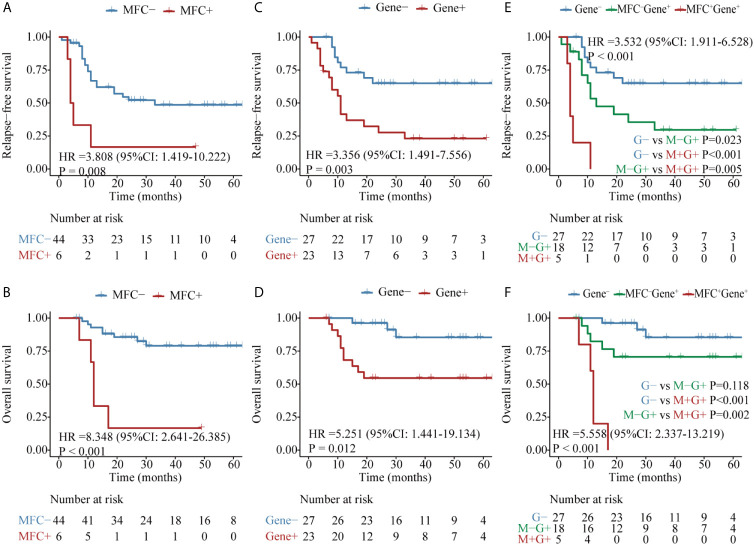
Kaplan–Meier curves for probability of relapse-free survival (RFS) and overall survival (OS) in AML patients with *RUNX1–RUNX1T1* fusion gene. **(A, B)** RFS and OS based on MFC MRD status post two cycles of consolidation. **(C, D)** RFS and OS based on the quantification of *RUNX1–RUNX1T1* transcript levels post two cycles of consolidation. **(E, F)** RFS and OS based on the integration of the two MRD monitoring methods.

**Figure 3:**
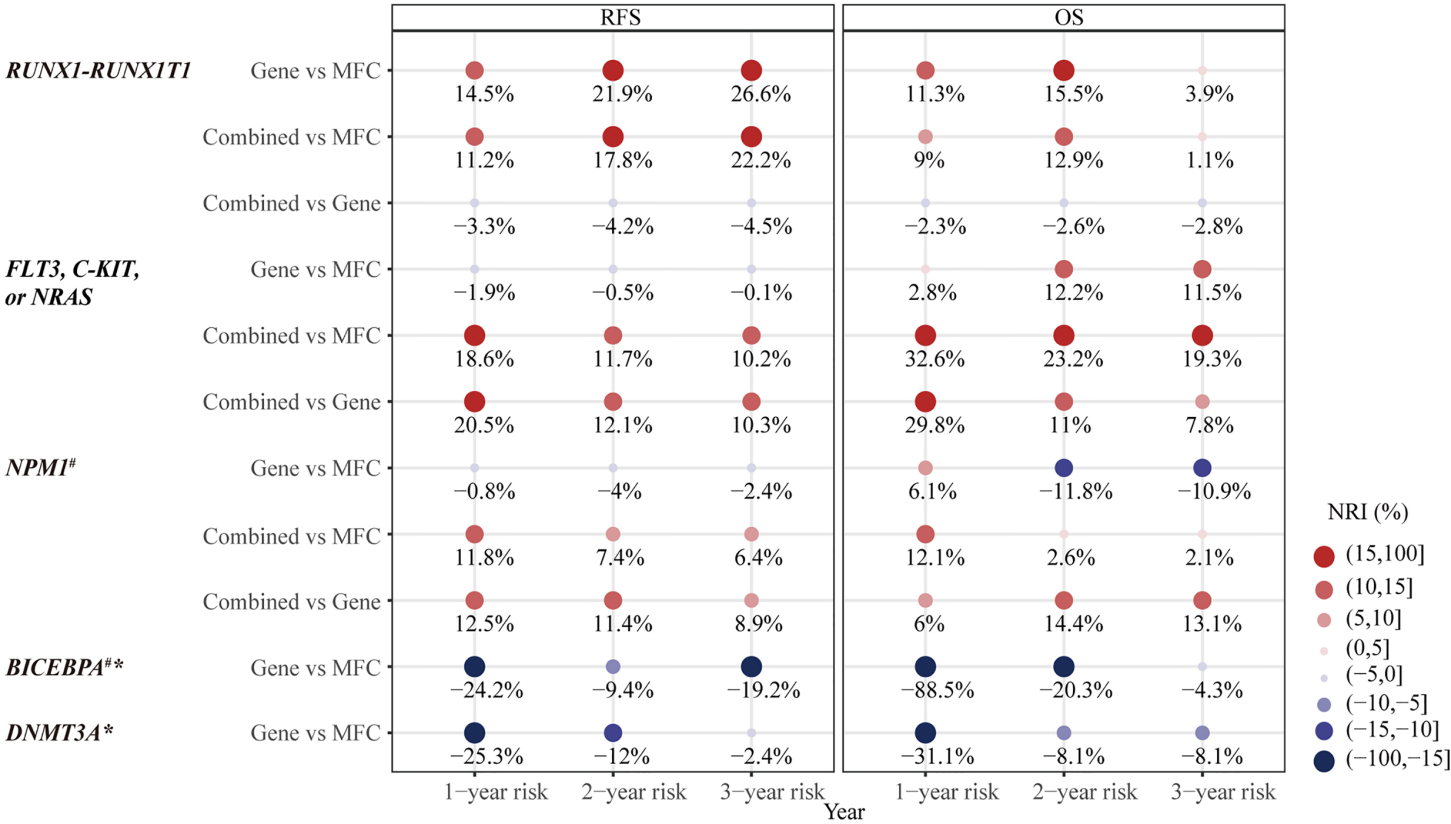
The category-based net reclassification improvement (NRI) of prediction on relapse-free survival (RFS) and overall survival (OS) by MRD status in different types of AML-related genetic lesions. ^#^Notice, there were less than three people in Gene^+^ group. *no significant difference in prognosis predicted by Gene MRD and therefore no combination of MFC MRD and Gene MRD. NRI, net reclassification improvement; MFC, multicolor flow cytometry; MRD, measurable residual disease; Combined MRD, MRD positivity detected by either MFC or molecular method; RFS, relapse-free survival; OS, overall survival.

Firstly, the two approaches were compared in 50 patients who harbored the *RUNX1*–*RUNX1T1* fusion gene at diagnosis. Both MFC MRD status (median RFS, 33 *vs* 5 months, P = 0.008, median OS, NR *vs* 12 months, P <0.001) (**Figures 2A, B**) and molecular MRD status (median RFS, NR *vs* 11 months, P = 0.003; median OS, NR, P = 0.012) **Figures 2C, D** could distinguish patients with a relatively favorable outcome from those with an increased risk of relapse and mortality, while the presence of both molecular and MFC MRD indicated the worst prognosis (**Figures 2E, F**). Of note, the NRI of molecular MRD in 2-year RFS and OS was 21.9 and 15.5%, respectively, compared with MFC MRD (**Figure 3**), and more importantly, there was no improvement when two methods were combined.

**Figure 4:**
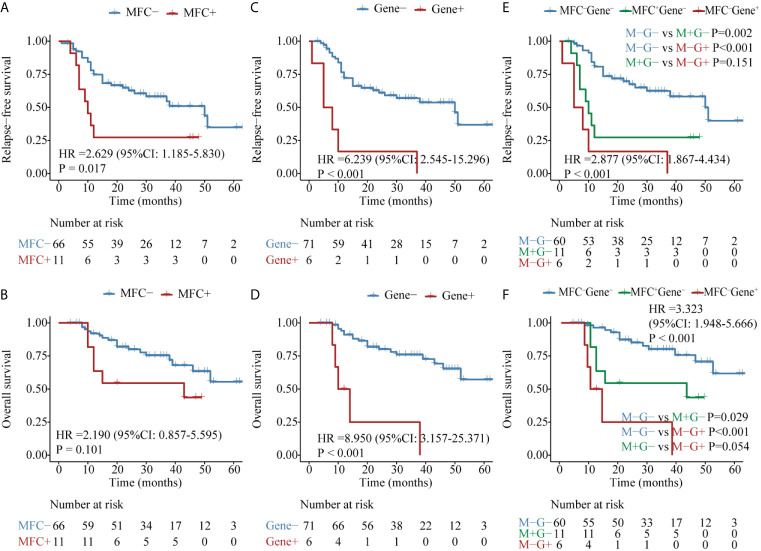
Kaplan–Meier curves for probability of relapse-free survival (RFS) and overall survival (OS) in AML patients with kinase-related signaling pathway mutations. **(A, B)** RFS and OS based on MFC MRD status post two consolidation cycles. **(C, D)** RFS and OS based on *FLT3*, *C-KIT*, or *N-RAS* mutations status by post two consolidation cycles. **(E, F)** RFS and OS based on MFC MRD status and Gene MRD status post two consolidation cycles.

The second panel of genes was involved in activated signaling pathways, and 77 patients with *FLT3*, *C-KIT*, or *N-RAS* mutations at diagnosis were evaluated. Both MFC MRD status (median, 50 *vs* 10 months, P = 0.017) (**Figure 4A**) and molecular MRD status (median, 50 *vs* 7 months, P <0.001) (**Figure 4C**) had a significant prognostic impact on RFS. Gene MRD positivity conferred a significantly worse OS (median, NR *vs* 12 months, P <0.001, **Figure 4D**), while the presence of MFC MRD was borderline associated with an inferior OS (median, NR *vs* 43 months, P = 0.101, **Figure 4B**). Patients with MFC^–^Gene^–^ had longer RFS and OS than those in either MFC^+^Gene^–^ or MFC^–^Gene^+^ group (P <0.001, P = 0.002 for RFS; P <0.001, P = 0.029 for OS, respectively, **Figures 4E, F**). The NRI showed positive gains in reclassification when combing MFC and Gene MRD together, with 11.7 and 23.2% improved value in the prediction of 2-year RFS and OS, respectively, compared with MFC MRD status, and the improvement was 12.1 and 11%, respectively, compared with molecular MRD method (**Figure 3**).

**Figure 5:**
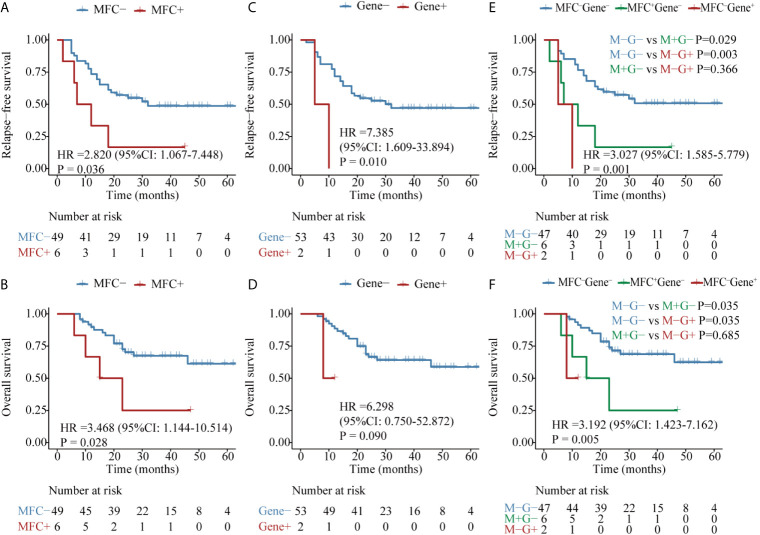
Kaplan–Meier curves for probability of relapse-free survival (RFS) and overall survival (OS) in AML patients with *NPM1* mutations. **(A, B)** RFS and OS based on MFC MRD status post-consolidation. **(C, D)** RFS and OS based on *NPM1* mutations status post two consolidation cycles. **(E, F)** RFS and OS based on MFC MRD status and Gene MRD status post two consolidation cycles.

Then, in 55 AML patients with *NPM1* mutations, the MFC MRD status after the second consolidation exerted a significant prognostic impact on both RFS (median RFS, 32 *vs* 10 months, P = 0.036) and OS (median OS, NR *vs* 19 months, P = 0.028) (**Figures 5A, B**). Only two patients harbored *NPM1* mutations post consolidation, and both experienced relapse within 10 months, one of whom succumbed to the disease, resulting in the shorter duration of both RFS (median RFS,30 *vs* 8 months, P = 0.010) and OS (median OS, NR *vs* 8 months, P = 0.090) compared with patients who had a clearance of *NPM1* mutations (**Figures 5C, D**). Patients with MFC^–^Gene^–^ obtained significantly longer RFS and OS than those with either MFC or Gene MRD positive (all P <0.05, **Figures 5E, F**). The integration of both assays yielded 7.4 and 11.4% NRI for 2-year RFS compared to the MRD status evaluated by MFC and *NPM1* mutations, respectively, and 14.4% for 2-year OS compared to Gene MRD (**Figure 3**).

Patients with Bi*CEBPA* mutations were reported to be sensitive to standard chemotherapy. Consistently, there were only six patients in the MFC MRD^+^ group and 1 patient in the Gene MRD^+^ group after two cycles of consolidation. MFC MRD positivity tended to predict a worse RFS (P = 0.013, **Figure S8A**) but did not impact OS (P = 0.745, **Figure S8B**). The only patient with Gene MRD^+^ experienced a long-term survival, therefore no significant differences were observed in RFS and OS between different Gene MRD groups (**Figures S8C, D**).

For 25 patients carrying mutations in *DNMT3A*, the elimination of MFC MRD was significantly associated with longer RFS (P = 0.011) and OS (P = 0.049) (**Figures S9A, B**), while no significant differences were observed in RFS (P = 0.902) and OS (P = 0.596) between patients with detectable *DNMT3A* mutations and those whose molecular MRD turned into negative (**Figures S9C, D**).

## Discussion

There is now mounting evidence that the identification of residual disease is of paramount importance in refining risk reclassification and informing therapeutic intervention for AML patients after the achievement of morphological remission (16, 22, 23). However, no consensus has been reached on the ideal time point and the optimal methodology for MRD evaluation, highlighting the need to establish standardized analysis and reporting procedures so as to improve the accuracy and reproducibility of MRD monitoring.

Our results indicated that the MFC MRD status after two consolidation cycles had a greater impact on the subsequent relapse and inferior outcome than that measured after induction. Notably, the same conclusion could be drawn when restricting the analysis to different patient subgroups (young *vs.* elderly patients, and ELN cytogenetic low- or intermediate-risk patients). While for high-risk patients, the MFC MRD status at both time points failed to forecast the prognosis, which merits further exploration considering that adverse molecular markers, e.g., *TP53*, *ASXL1*, and *RUNX1* were not included in our study. Our results are consistent with previous studies that recommended MRD tracking after consolidation (9, 13, 24), although others favored the post-induction time point (25). It should be mentioned that the controversial interpretation of the prognostic value of MRD in different studies may be attributed to the number of induction and consolidation courses completed at the time of MFC MRD monitoring, and the modality and intensity of induction regimens, as reported by Minetto et al. for fludarabine plus high dose cytarabine-based induction, an earlier timepoint of MRD assessment may provide the most significant information on outcome (26).

The dynamics of sequential MRD monitoring demonstrated that patients who had detectable MRD after induction but entered MRD negativity after the second consolidation showed the same prognosis as those with a negative MRD at both time points. In contrast, the initial clearance of MRD did not guarantee a persistent remission and long-term survival, as exemplified by the dismal prognosis of patients whose MRD was eliminated early and subsequently converted into positive after consolidation. More importantly, the achievement of MFC MRD negativity after two consolidation cycles was an independent predictor for both RFS and OS, emphasizing the need to introduce new therapeutic modalities such as HSCT and targeted therapies to eradicate residual malignant cells when MFC MRD was positive at this checkpoint.

In addition to abnormal immunophenotypes detected by MFC, MRD could be reliably measured through genetic aberrations expressed in leukemic cells. We compared the Gene MRD and MFC MRD after the second consolidation in several molecular groups representative of different genetic etiology and biological function. Gene fusions involving transcription factors such as *RUNX1–RUNX1T1* represent a specific subtype of AML. Although significantly diverse prognostic groups could be distinguished by both MFC and molecular MRD, the latter methodology was superior in terms of the NRI. Indeed, a less than 3 log reduction in the *RUNX1–RUNX1T1* transcript levels was proved to be an independent adverse prognostic factor (23, 27).

Mutations in kinase-related signaling pathways including *FLT3, CKIT*, or *N-RAS* mutations usually occur at a later stage and are more likely to be eliminated by cytotoxic chemotherapy. The clearance of MRD confirmed by either MFC or sequencing-based approach conferred a favorable clinical outcome, and the combination of both methods showed greater discriminative ability. Similar results could be observed in patients with *NPM1* mutations. Although only two patients harbored NPM1 mutations post consolidation, both displayed a dismal prognosis, which was in concordance with the widely appreciated role of *NPM1* mutations in MRD testing (28, 29). Remarkably, the MFC MRD can provide complementary prognostic value.

Biallelic *CEBPA* mutations have been recognized as a favorable prognostic marker of AML (18, 30, 31). However, since only one patient was in the Gene MRD^+^ group after consolidation, the tracking of molecular MRD showed limited predictive power as reported in prior studies (32, 33). Likewise, the continuous presence of mutant transcripts in the epigenetic modifier gene *DNMT3A* did not exert any adverse impact on prognosis. Consistently, recent researches regarded *DNMT3A*, *TET2*, and *ASXL1* (DTA) mutations as age-related clonal hematopoiesis and their persistent existence post remission was unable to forecast an increased risk of relapse (3, 34).

Despite the high performance of MRD monitoring by MFC and PCR-based assays, a proportion of AML patients lack a traceable MRD marker. So far, routine clinical practices of MRD tracking have dealt with only a small proportion of typical genetic anomalies in AML (5, 35). The overexpression of *WT1* can be observed in more than 80% of AML patients, which may be an alternative PCR-based MRD testing since the quantification of *WT1* expression after treatment has been proved to have significant prognostic value (36). In addition, the integration of *WT1*-based MRD and MFC MRD may improve the prediction of outcome in AML, although the limited sensitivity and specificity to some extent hamper the wide application of MRD monitoring based on *WT1* expression (37–39). Encouragingly, the NGS technology holds great potential for the widely applicable MRD tracking as nearly all AML patients harbored at least one mutation at diagnosis. Growing evidence has proved the prognostic value of NGS-based MRD, either in the CR or pre-transplant stage, which can provide additional information on changes of variant allele frequency as well as clonal evolution during the follow-up period (3, 40–42). However, these NGS-based studies often integrated dozens to hundreds of genetic abnormalities without uniform design, technical and reporting standards, which may ignore the heterogeneity of molecular anomalies and their utility for MRD tracking in AML (40). It is noteworthy that the predictive value of molecular MRD varies in different categories of mutations as manifested in this study, and the optimal threshold of NGS-based MRD needs to be explored in a flexible and genotype-oriented way.

In summary, our study indicated that a positive MFC MRD after two consolidation cycles was an independent risk factor, and the comparison of molecular and MFC MRD in patients with different types of recurrent mutations lends support to the clinical implementation of NGS-based MRD assessment. Due to the limitation of technologies, a few germline mutations could affect the explanation of molecular MRD. Hence, how to integrate various detection methodologies and establish standard-of-care guidelines for MRD testing warrants further refinements in large prospective studies.

## Data Availability Statement

The original contributions presented in the study are included in the article/**Supplementary Material**. Further inquiries can be directed to the corresponding author.

## Ethics Statement

The studies involving human participants were reviewed and approved by Ruijin Hospital Ethics Committee Shanghai Jiao Tong University School of Medicine. Written informed consent to participate in this study was provided by the participants’ legal guardian/next of kin.

## Author Contributions

F-JL, W-YC, X-JL, and S-YW contributed equally to this work. F-JL, W-YC, S-YW, and T-YJ collected clinical data. Y-MZ designed the experiments. Y-MZ and T-TM performed the experiments. F-JL and X-JL analyzed the data. F-JL and W-YC wrote the manuscript. YS conceived the overall study and revised the paper. All authors contributed to the article and approved the submitted version.

## Funding

This work was supported in part by the National Natural Science Foundation of China (no. 81770141), the National Key R&D Program of China (no. 2016YFE0202800), and Shanghai Municipal Education Commission-Gaofeng Clinical Medicine Grant Support (no. 20161406).

## Conflict of Interest

The authors declare that the research was conducted in the absence of any commercial or financial relationships that could be construed as a potential conflict of interest.
