# Supplementary material for: Measurable Residual Disease Detected by Multiparameter Flow Cytometry and Sequencing Improves Prediction of Relapse and Survival in Acute Myeloid Leukemia
Source: Front Oncol. 2021 May 20;11:677833. doi: 10.3389/fonc.2021.677833 (PMC8173083; doi:10.3389/fonc.2021.677833)
Supplement: Supplementary file 1 [file DataSheet_1.pdf]

Supplementary Material

1 Supplementary Methods

1.1 Treatment protocols

In the three clinical trials, the number of peripheral blast cells in patients with acute myeloid leukemia (AML) were monitored by the multiparameter flow cytometry (MFC) at two time points (D0 and D5) of the standard “3+7” induction chemotherapy, by which the D5 peripheral blast clearance rate (D5-PBCR) was calculated. In the phase II clinical trial (ChiCTR-OPC-15006085) designed for young patients (<55 years), homoharringtonine (HHT, 2mg/m^2, D6-10) was prescribed in those with D5-PBCR <99.55% and HHT was used at random for patients (18-60 years) with D5-PBCR <99.55% in the phase III clinical trial (ChiCTR-OIC-16007764). For patients (60-75 years) enrolled in the other phase II trial (ChiCTR-OIN-16008955), additional doses of idarubicin (6mg/m^2, D6-7) were administered when the D5-PBCR was <99.55%. If complete remission was achieved, consolidation therapy was based on the risk stratification of patients, using either high-dose cytarabine-containing chemotherapy or hematopoietic stem cell transplantation. When patients attained partial remission, another cycle of the same induction regimens was given before consolidation.

1.2 Definitions in multiparameter flow cytometry

MFC data were acquired using a NAVIOS cytometer (Beckman Coulter, Brea, California) and analyzed by KALUZA software (Beckman Coulter, Brea, California). The leukemia-associated immunophenotype (LAIP) was identified as cell populations showing completely different patterns of antigenic expression from either normal or regenerating BM counterparts at specific stage maturation of myeloid precursors. Abnormal immunophenotypes consisted of four main types, namely, cross-lineage antigen expression, asynchronous antigen expression, antigen dim/strong expression, and antigen expression missing. The MFC MRD was defined as at least 20 clustered cells showing myeloid-scattering properties and LAIP characteristics, and a minimum of 1×10^6 white blood cells (WBCs) were required for MRD monitoring to achieve a theoretically maximum sensitivity of 2×10^{-5}.

1.3 Definitions of responses and outcome endpoints

Complete remission (CR) was defined as less than 5% blasts in BM by cytomorphology accompanied with the recovery of absolute neutrophil count (>1×10^9/L) and platelet count (>100×10^9/L) in peripheral blood. Relapse-free survival (RFS) was measured from the time of the first achievement of CR to relapse or death, whichever occurred first, and patients who lost contact or alive in CR at the last follow-up were censored. Overall survival (OS) was measured from the date of disease diagnosis to death from any cause, and patients alive were censored at the time of the last follow-up. Median follow-up was calculated using the reverse Kaplan-Meier method.

2 Supplementary Tables

Table S1. Genetic alteration patterns of AML patients.

Variable No./Total (%)	De Novo AML (n =639)	Without LAIPs （n = 52）	With LAIPs (n = 587)	P
**Genetic Alterations**				
RUNX1-RUNX1T1	84/572 (14.7)	1/43 (2.3)	83/529 (15.7)	0.031
CBFβ-MYH11	37/547 (6.8)	7/38 (18.4)	30/509 (5.9)	0.009
FLT3-ITD	60/588 (10.2)	6/45 (13.3)	54/543 (9.9)	0.642
FLT3-TKD	30/586 (5.1)	1/44 (2.3)	29/542 (5.4)	0.592
KMT2A-fusion	29/586 (4.9)	0/45 (0.0)	29/541 (5.4)	0.217
KMT2A-AF9	12/586 (2.0)	0/45 (0.0)	12/541 (2.2)	0.644
KMT2A-PTD	25/585 (4.3)	4/44 (9.1)	21/541 (3.9)	0.209
NPM1	127/581 (21.8)	15/44 (34.1)	112/539 (20.8)	0.062
CKIT	64/568 (11.3)	4/41 (9.8)	60/507 (11.4)	0.951
NRAS	84/572 (14.7)	1/43 (2.3)	83/529 (15.7)	0.597
BiCEBPA	111/586 (18.9)	0/45 (0.0)	111/541 (20.5)	0.001
DNMT3A	68/586 (11.6)	9/46 (19.6)	59/540 (10.9)	0.129

LAIPs: leukemia-associated immunophenotypes

Table S2. The eight most common LAIPs in AML patients.

LAIP subtypes	No. of cases	Frequency
CD7+CD34+CD117+CD13+CD33+CD45dimSSClow	152	25.89%
CD56+CD34+CD117+CD45dimSSClow	106	18.06%
CD19+CD34+CD117+CD45dimSSClow	44	7.50%
CD7+CD117+CD13+CD33+CD45dimSSClow	25	4.26%
CD34+CD117+CD13+CD33+HLA-DR-CD45dimSSClow	15	2.56%
CD7+CD34-CD117+CD13+CD33+CD45dimSSClow	13	2.21%
CD7+CD34+CD117+CD13+CD45dimSSClow	13	2.21%
CD7+CD34+CD117+CD33+CD45dimSSClow	13	2.21%

LAIPs: leukemia-associated immunophenotypes

Table S3. Survival data among different MFC MRD groups post induction and post two cycles of consolidation

MRD groups		RFS		OS	
		RFS Probability (3-year) (95% CI)	Median RFS (months) (95% CI)	OS Probability (3-year) (95% CI)	Median OS (months) (95% CI)
Post-induction	MFC^{PI-} (n=329)	0.486 (0.432-0.547)	32 (26-NR)	0.710 (0.659-0.764)	NR
	MFC^{PI+} (n=163)	0.362 (0.293-0.446)	13 (10-20)	0.553 (0.477-0.640)	50 (30-NR)
Post-consolidation	MFC^{PC-} (n=341)	0.550 (0.497-0.609)	67 (36-NR)	0.765 (0.718-0.815)	NR
	MFC^{PC+} (n=80)	0.293 (0.207-0.416)	16 (9-20)	0.521 (0.419-0.649)	43 (20-NR)
Post-induction / Post-consolidation	$MFC^{PI-}MFC^{PC-}$ (n=274)	0.546 (0.486-0.613)	67 (33-NR)	0.770 (0.718-0.826)	NR
	$MFC^{PI+}MFC^{PC-}$ (n=67)	0.552 (0.443-0.688)	56 (19-NR)	0.743 (0.642-0.861)	NR
	$MFC^{PI+}MFC^{PC+}$ (n=47)	0.342 (0.226-0.517)	16 (10-38)	0.584 (0.453-0.753)	50 (22-NR)
	$MFC^{PI-}MFC^{PC+}$ (n=33)	NR	8 (6-15)	NR	21 (17-NR)

MFC, multicolor flow cytometry; MRD, measurable residual disease; RFS, relapse-free survival; OS, overall survival; NR, not reached; CI, confidence interval; MFC^{PI-}, MFC MRD negative post induction; MFC^{PI+}, MFC MRD positive post induction; MFC^{PC-}, MFC MRD negative post two cycles of consolidation; MFC^{PC+}, MFC MRD positive post two cycles of consolidation.

Table S4. Univariate Analysis of Prognostic Variables for RFS and OS.

Variables	RFS		OS	
	HR (95% CI)	P	HR (95% CI)	P
Age	1.021(1.012-1.029)	<0.001	1.025(1.015-1.036)	<0.001
Gender	1.083(0.852-1.377)	0.515	1.186(0.880-1.597)	0.262
WBC count at diagnosis($\times 10^9$/L) (>100 vs ≤100)	1.195(0.758-1.884)	0.444	1.003(1.000-1.006)	0.040
HB at diagnosis	0.994(0.990-0.999)	0.013	0.994(0.988-0.999)	0.028
PLT count at diagnosis	1.000(0.998-1.002)	0.701	1.001(0.999-1.003)	0.374
BM Blasts	1.002(0.996-1.007)	0.540	1.006(0.999-1.013)	0.096
2017 ELN cytogenetic stratification				
Intermediate vs Favorable	1.677 (1.273-2.209)	<0.001	1.890(1.346-2.654)	<0.001
Unfavorable vs Favorable	1.293 (1.051-1.591)	0.015	1.251(0.970-1.614)	0.085
FLT3-ITD	1.652(1.092-2.499)	0.017	2.262(1.442-3.547)	<0.001
FLT3-TKD	1.236(0.674-2.264)	0.493	1.247(0.584-2.665)	0.568
KMT2A-PTD	1.363(0.700-2.654)	0.362	1.703(0.798-3.635)	0.169
CKIT	1.451(0.999-2.108)	0.051	1.776(1.157-2.727)	0.009
NRAS	0.839(0.587-1.200)	0.337	0.705(0.441-1.127)	0.144
NPM1	1.138(0.849-1.526)	0.386	1.250(0.876-1.783)	0.218
Bi*CEBPA*	0.440(0.308-0.630)	<0.001	0.339(0.207-0.554)	<0.001
DNMT3A	1.231(0.857-1.769)	0.261	1.336(0.866-2.061)	0.191
CR achieved: 2 cycles vs 1 cycle	1.607(1.160-2.228)	0.004	1.628(1.099-2.412)	0.015
HSCT *	0.935(0.720-1.214)	0.613	0.717(0.511-1.007)	0.055
MFC^{PI+} vs MFC^{PI-}	1.659(1.299-2.119)	<0.001	1.738(1.287-2.345)	<0.001
MFC^{PC+} vs MFC^{PC-}	2.323(1.711-3.153)	<0.001	2.205(1.511-3.218)	<0.001

* patients accepted HSCT after the first two consolidation were included in multivariate models for RFS and OS. RFS, relapse-free survival; OS, overall survival. HR, Hazard ratio; CI, confidence interval; WBC, white blood cell; HB, hemoglobin; PLT, platelet; BM, bone marrow; HSCT, hematopoietic stem cell transplantation; MRD, measurable residual disease; MFC, multicolor flow cytometry; MRD, measurable residual disease; MFC^{PI+}, MFC MRD positive post induction; MFC^{PI-}, MFC MRD negative post induction; MFC^{PC+}, MFC MRD positive post two cycles of consolidation; MFC^{PC-}, MFC MRD negative post two cycles of consolidation.

Table S5. Univariate Analysis of Prognostic Variables for RFS and OS in 2017 ELN cytogenetics intermediate risk group

Variables	RFS		OS	
	HR (95% CI)	P	HR (95% CI)	P
Age	1.022(1.010-1.033)	<0.001	1.028(1.013-1.043)	<0.001
Gender	1.111(0.801-1.540)	0.529	1.074(0.720-1.603)	0.725
WBC count at diagnosis(×10^9/L) (>100 vs ≤100)	1.565(0.846-2.895)	0.154	2.320(1.204-4.471)	0.012
HB at diagnosis	0.995(0.989-1.001)	0.122	0.996(0.989-1.004)	0.295
PLT count at diagnosis	1.000(0.998-1.003)	0.981	1.001(0.998-1.004)	0.422
BM Blasts	1.001(0.994-1.009)	0.687	1.007(0.998-1.016)	0.130
FLT3-ITD	2.210(1.344-3.633)	0.002	3.006(1.748-5.170)	<0.001
FLT3-TKD	1.171(0.479-2.864)	0.729	0.757(0.186-3.078)	0.697
KMT2A-PTD	1.150(0.366-3.613)	0.811	1.685(0.533-5.328)	0.375
CKIT	1.281(0.472-3.476)	0.627	1.159(0.284-4.730)	0.838
NRAS	0.951(0.579-1.562)	0.843	0.837(0.445-1.573)	0.580
NPM1	1.458(1.026-2.073)	0.035	1.652(1.079-2.531)	0.021
Bi*CEBPA*	0.503(0.341-0.742)	0.001	0.373(0.222-0.627)	<0.001
DNMT3A	1.266(0.820-1.955)	0.287	1.394(0.831-2.338)	0.208
CR achieved: 2 cycles vs 1 cycle	1.244(0.759-2.038)	0.386	1.613(0.930-2.798)	0.089
HSCT *	0.929(0.647-1.333)	0.688	0.838(0.534-1.314)	0.441
MFC^{PI+} vs MFC^{PI-}	1.754(1.262-2.438)	0.001	1.672(1.116-2.504)	0.013
MFC^{PC+} vs MFC^{PC-}	2.224(1.466-3.373)	<0.001	1.815(1.072-3.074)	0.027

* patients accepted HSCT after the first two consolidation were included in multivariate models for RFS and OS. RFS, relapse-free survival; OS, overall survival. HR, Hazard ratio; CI, confidence interval; WBC, white blood cell; HB, hemoglobin; PLT, platelet; BM, bone marrow; HSCT, hematopoietic stem cell transplantation; MRD, measurable residual disease; MFC, multicolor flow cytometry; MRD, measurable residual disease; MFC^{PI+}, MFC MRD positive post induction; MFC^{PI-}, MFC MRD negative post induction; MFC^{PC+}, MFC MRD positive post two cycles of consolidation; MFC^{PC-}, MFC MRD negative post two cycles of consolidation.

Table S6. Multivariate Analysis of Prognostic Variables for RFS and OS in 2017 ELN cytogenetics intermediate risk group

Variables	RFS		OS	
	HR (95% CI)	P	HR (95% CI)	P
Age	1.022(1.006-1.038)	0.006	1.026(1.006-1.046)	0.009
WBC at diagnosis($\times 10^9$/L) (>100 vs ≤100)	2.146(0.947-4.860)	0.067	2.749(1.105-6.838)	0.030
FLT3-ITD	1.833(0.982-3.420)	0.057	2.549(1.293-5.027)	0.007
NPM1	1.217(0.755-1.962)	0.420	1.511(0.833-2.743)	0.175
Bi*CEBPA*	0.432(0.245-0.760)	0.004	0.416(0.200-0.865)	0.019
CR achieved: 2 cycles vs 1 cycle	0.774(0.405-1.480)	0.439	1.021(0.492-2.122)	0.955
MFC^{PI+} vs MFC^{PI-}	1.264(0.801-1.994)	0.314	1.399(0.796-2.461)	0.243
MFC^{PC+} vs MFC^{PC-}	3.847(2.281-6.489)	<0.001	3.582(1.882-6.816)	<0.001

RFS, relapse-free survival; OS, overall survival. HR, Hazard ratio; CI, confidence interval; WBC, white blood cell; CR, complete remission; MRD, measurable residual disease; MFC^{PI+}, MFC MRD positive post induction; MFC^{PI-}, MFC MRD negative post induction; MFC^{PC+}, MFC MRD positive post two cycles of consolidation. MFC^{PC-}, MFC MRD negative post two cycles of consolidation.

3 Supplementary Figures

Supplementary Figure 1. Patient flow diagram

Supplementary Figure 2. Kaplan-Meier curves for probability of relapse-free survival (RFS) and overall survival (OS) in AML patients. RFS and OS based on MFC MRD status at a threshold of 0% post induction (A, B), and post two consolidation cycles (C, D). RFS and OS based on MFC MRD status at a threshold of 0.01% post induction (E, F), and post two consolidation cycles (G, H). RFS and OS based on MFC MRD status at a threshold of 0.035% post induction (I, J), and post two consolidation cycles (K, L). RFS and OS based on MFC MRD status at a threshold of 1% post induction (M, N), and post two consolidation cycles (O, P).

Supplementary Figure 3 Kaplan-Meier curves for probability of relapse-free survival (RFS) and overall survival (OS) in young AML patients (< 60 y). (A, B) RFS and OS based on MFC MRD status post induction. (C, D) RFS and OS based on MFC MRD status post two consolidation cycles. (E, F) RFS and OS based on MFC MRD status in the combination of both time points.

Supplementary Figure 4 Kaplan-Meier curves for probability of relapse-free survival (RFS) and overall survival (OS) in old AML patients (≥ 60 y). (A, B) RFS and OS based on MFC MRD status post induction. (C, D) RFS and OS based on MFC MRD status post two consolidation cycles. (E, F) RFS and OS based on MFC MRD status in the combination of both time points.

Supplementary Figure 5 Kaplan-Meier curves for probability of relapse-free survival (RFS) and overall survival (OS) in AML patients with low-risk cytogenetics. (A, B) RFS and OS based on MFC MRD status post induction. (C, D) RFS and OS based on MFC MRD status post two consolidation cycles. (E, F) RFS and OS based on MFC MRD status in the combination of both time points.

Supplementary Figure 6 Kaplan-Meier curves for probability of relapse-free survival (RFS) and overall survival (OS) in AML patients with intermediate-risk cytogenetics. (A, B) RFS and OS based on MFC MRD status post induction. (C, D) RFS and OS based on MFC MRD status post two consolidation cycles. (E, F) RFS and OS based on MFC MRD status in the combination of both time points.

Supplementary Figure 7 Kaplan-Meier curves for probability of relapse-free survival (RFS) and overall survival (OS) in AML patients with high-risk cytogenetics. (A, B) RFS and OS based on MFC MRD status post induction. (C, D) RFS and OS based on MFC MRD status post two consolidation cycles. (E, F) RFS and OS based on MFC MRD status in the combination of both time points.

Supplementary Figure 8 Kaplan-Meier curves for probability of relapse-free survival (RFS) and overall survival (OS) in AML patients with biallelic mutations of *CEBPA*. (A, B) RFS and OS based on MFC MRD status post two cycles of consolidation. (C, D) RFS and OS based on biallelic *CEBPA* mutations post two cycles of consolidation.

Supplementary Figure 9 Kaplan-Meier curves for probability of relapse-free survival (RFS) and overall survival (OS) in AML patients with *DNMT3A* mutations. (A, B) RFS and OS based on MFC MRD status post two cycles of consolidation. (C, D) RFS and OS based on mutant *DNMT3A* transcripts post two cycles of consolidation.

Fig.S1 Patient flow diagram

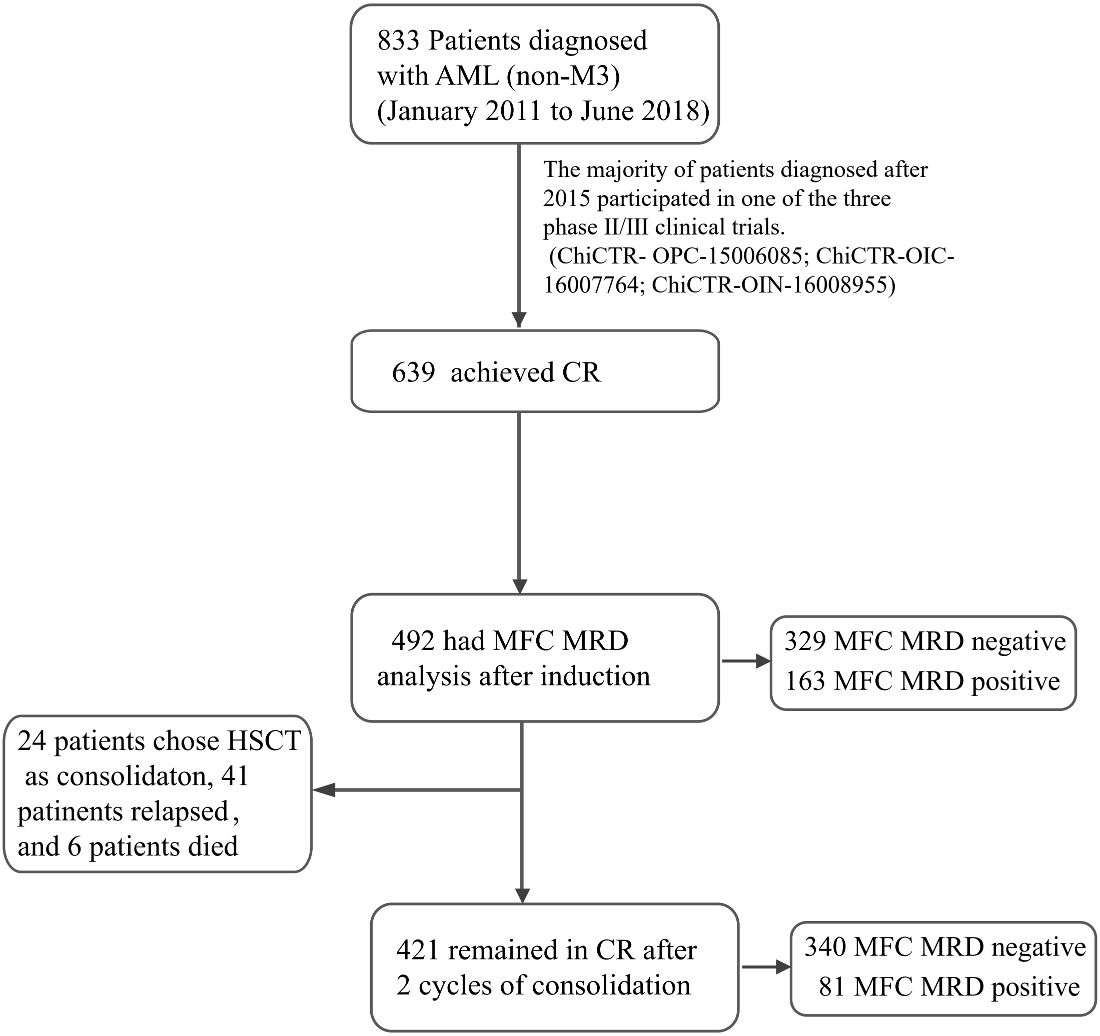

Fig.S2

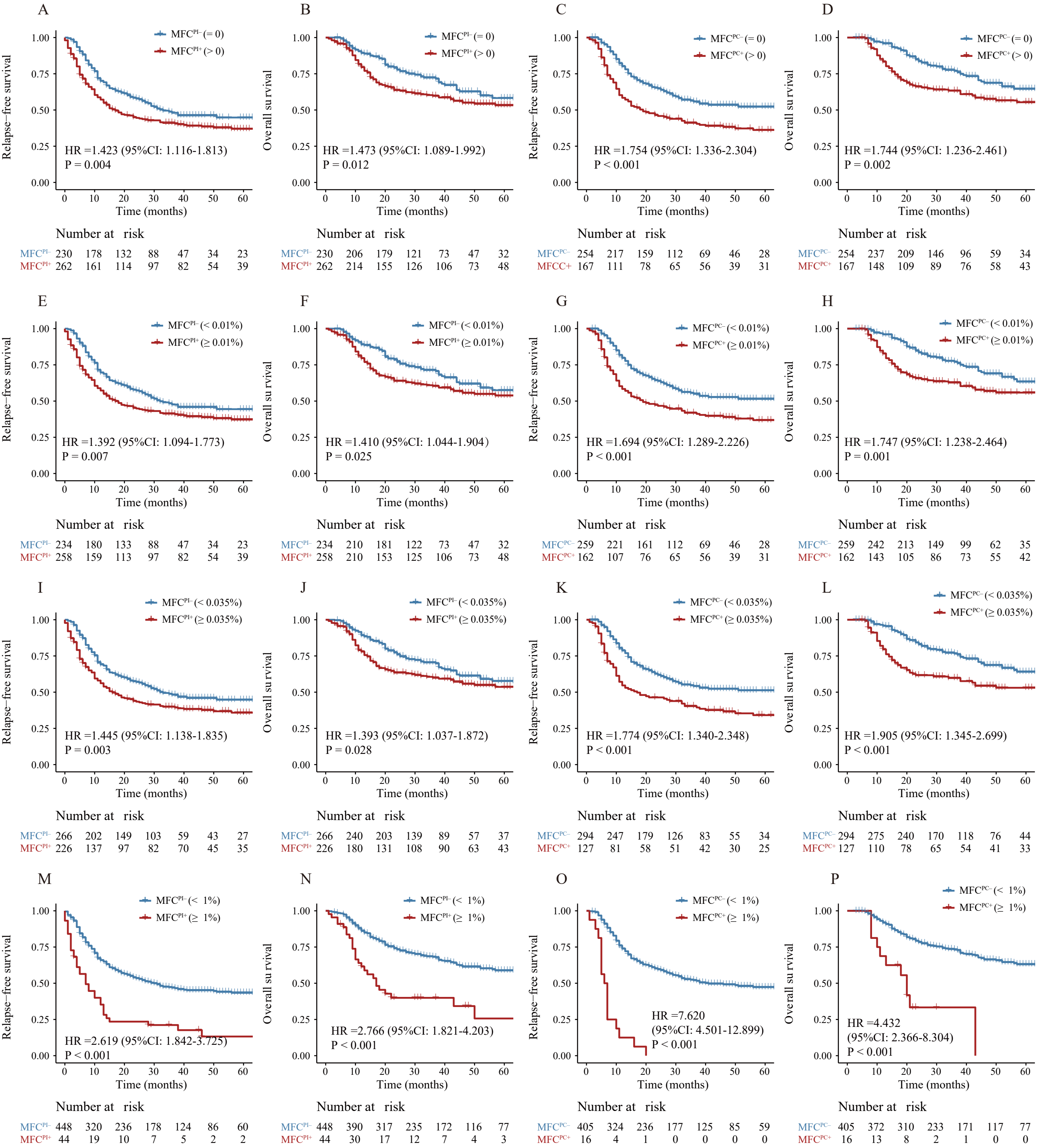
A
MFC^PI−(= 0)
MFC^PI+(> 0)
Relapse−free survival
HR =1.423 (95%CI: 1.116-1.813)
P = 0.004
Time (months)
Number at risk
MFC^PI− 230 178 132 88 47 34 23
MFC^PI+ 262 161 114 97 82 54 39
B
MFC^PI−(= 0)
MFC^PI+(> 0)
Overall survival
HR =1.473 (95%CI: 1.089-1.992)
P = 0.012
Number at risk
MFC^PI− 230 206 179 121 73 47 32
MFC^PI+ 262 214 155 126 106 73 48
C
MFC^PC−(= 0)
MFC^PC+(> 0)
HR =1.754 (95%CI: 1.336-2.304)
P < 0.001
MFC^PC− 254 217 159 112 69 46 28
MFCC+ 167 111 78 65 56 39 31
D
HR =1.744 (95%CI: 1.236-2.461)
P = 0.002
MFC^PC− 254 237 209 146 96 59 34
MFC^PC+ 167 148 109 89 76 58 43
E
MFC^PI−(< 0.01%)
MFC^PI+(≥ 0.01%)
HR =1.392 (95%CI: 1.094-1.773)
P = 0.007
MFC^PI− 234 180 133 88 47 34 23
MFC^PI+ 258 159 113 97 82 54 39
F
HR =1.410 (95%CI: 1.044-1.904)
P = 0.025
MFC^PI− 234 210 181 122 73 47 32
MFC^PI+ 258 210 153 125 106 73 48
G
MFC^PC−(< 0.01%)
MFC^PC+(≥ 0.01%)
HR =1.694 (95%CI: 1.289-2.226)
P < 0.001
MFC^PC− 259 221 161 112 69 46 28
MFC^PC+ 162 107 76 65 56 39 31
H
HR =1.747 (95%CI: 1.238-2.464)
P = 0.001
MFC^PC− 259 242 213 149 99 62 35
MFC^PC+ 162 143 105 86 73 55 42
I
MFC^PI−(< 0.035%)
MFC^PI+(≥ 0.035%)
HR =1.445 (95%CI: 1.138-1.835)
P = 0.003
MFC^PI− 266 202 149 103 59 43 27
MFC^PI+ 226 137 97 82 70 45 35
J
HR =1.393 (95%CI: 1.037-1.872)
P = 0.028
MFC^PI− 266 240 203 139 89 57 37
MFC^PI+ 226 180 131 108 90 63 43
K
MFC^PC−(< 0.035%)
MFC^PC+(≥ 0.035%)
HR =1.774 (95%CI: 1.340-2.348)
P < 0.001
MFC^PC− 294 247 179 126 83 55 34
MFC^PC+ 127 81 58 51 42 30 25
L
HR =1.905 (95%CI: 1.345-2.699)
P < 0.001
MFC^PC− 294 275 240 170 118 76 44
MFC^PC+ 127 110 78 65 54 41 33
M
MFC^PI−(< 1%)
MFC^PI+(≥ 1%)
HR =2.619 (95%CI: 1.842-3.725)
P < 0.001
MFC^PI− 448 320 236 178 124 86 60
MFC^PI+ 44 19 10 7 5 2 2
N
HR =2.766 (95%CI: 1.821-4.203)
P < 0.001
MFC^PI− 448 390 317 235 172 116 77
MFC^PI+ 44 30 17 12 7 4 3
O
MFC^PC−(< 1%)
MFC^PC+(≥ 1%)
HR =7.620
(95%CI: 4.501-12.899)
P < 0.001
MFC^PC− 405 324 236 177 125 85 59
MFC^PC+ 16 4 1 0 0 0 0
P
HR =4.432
(95%CI: 2.366-8.304)
P < 0.001
MFC^PC− 405 372 310 233 171 117 77
MFC^PC+ 16 13 8 2 1 0 0

Fig. S3

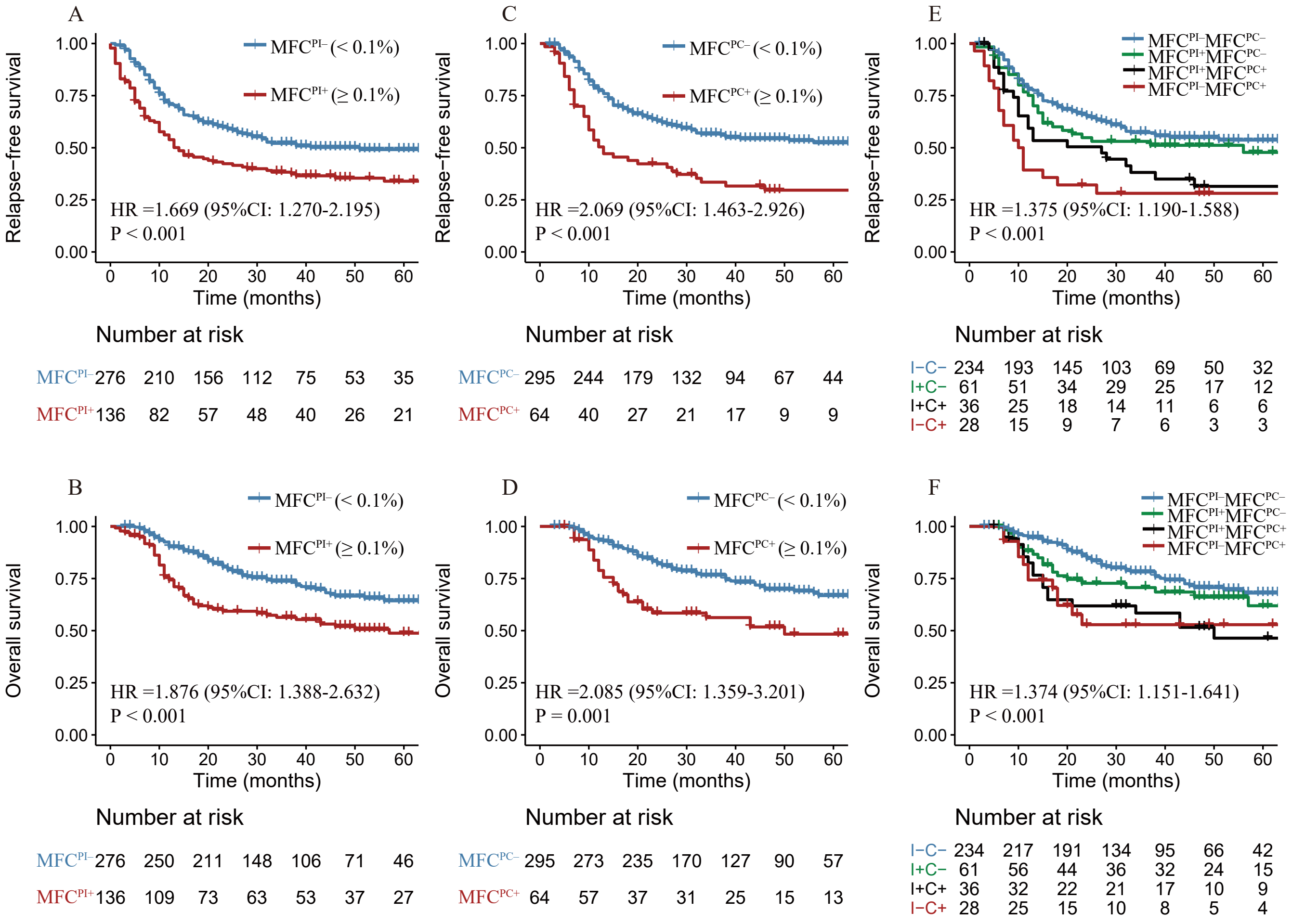
A
Relapse−free survival
MFC^PI−(< 0.1%)
MFC^PI+(≥ 0.1%)
HR =1.669 (95%CI: 1.270-2.195)
P < 0.001
Time (months)
Number at risk
MFC^PI− 276 210 156 112 75 53 35
MFC^PI+ 136 82 57 48 40 26 21
C
Relapse−free survival
MFC^PC−(< 0.1%)
MFC^PC+(≥ 0.1%)
HR =2.069 (95%CI: 1.463-2.926)
P < 0.001
Time (months)
Number at risk
MFC^PC− 295 244 179 132 94 67 44
MFC^PC+ 64 40 27 21 17 9 9
E
Relapse−free survival
MFC^PI−MFC^PC−
MFC^PI+MFC^PC−
MFC^PI+MFC^PC+
MFC^PI−MFC^PC+
HR =1.375 (95%CI: 1.190-1.588)
P < 0.001
Time (months)
Number at risk
I−C− 234 193 145 103 69 50 32
I+C− 61 51 34 29 25 17 12
I+C+ 36 25 18 14 11 6 6
I−C+ 28 15 9 7 6 3 3
B
Overall survival
MFC^PI−(< 0.1%)
MFC^PI+(≥ 0.1%)
HR =1.876 (95%CI: 1.388-2.632)
P < 0.001
Time (months)
Number at risk
MFC^PI− 276 250 211 148 106 71 46
MFC^PI+ 136 109 73 63 53 37 27
D
Overall survival
MFC^PC−(< 0.1%)
MFC^PC+(≥ 0.1%)
HR =2.085 (95%CI: 1.359-3.201)
P = 0.001
Time (months)
Number at risk
MFC^PC− 295 273 235 170 127 90 57
MFC^PC+ 64 57 37 31 25 15 13
F
Overall survival
MFC^PI−MFC^PC−
MFC^PI+MFC^PC−
MFC^PI+MFC^PC+
MFC^PI−MFC^PC+
HR =1.374 (95%CI: 1.151-1.641)
P < 0.001
Time (months)
Number at risk
I−C− 234 217 191 134 95 66 42
I+C− 61 56 44 36 32 24 15
I+C+ 36 32 22 21 17 10 9
I−C+ 28 25 15 10 8 5 4

Fig. S4

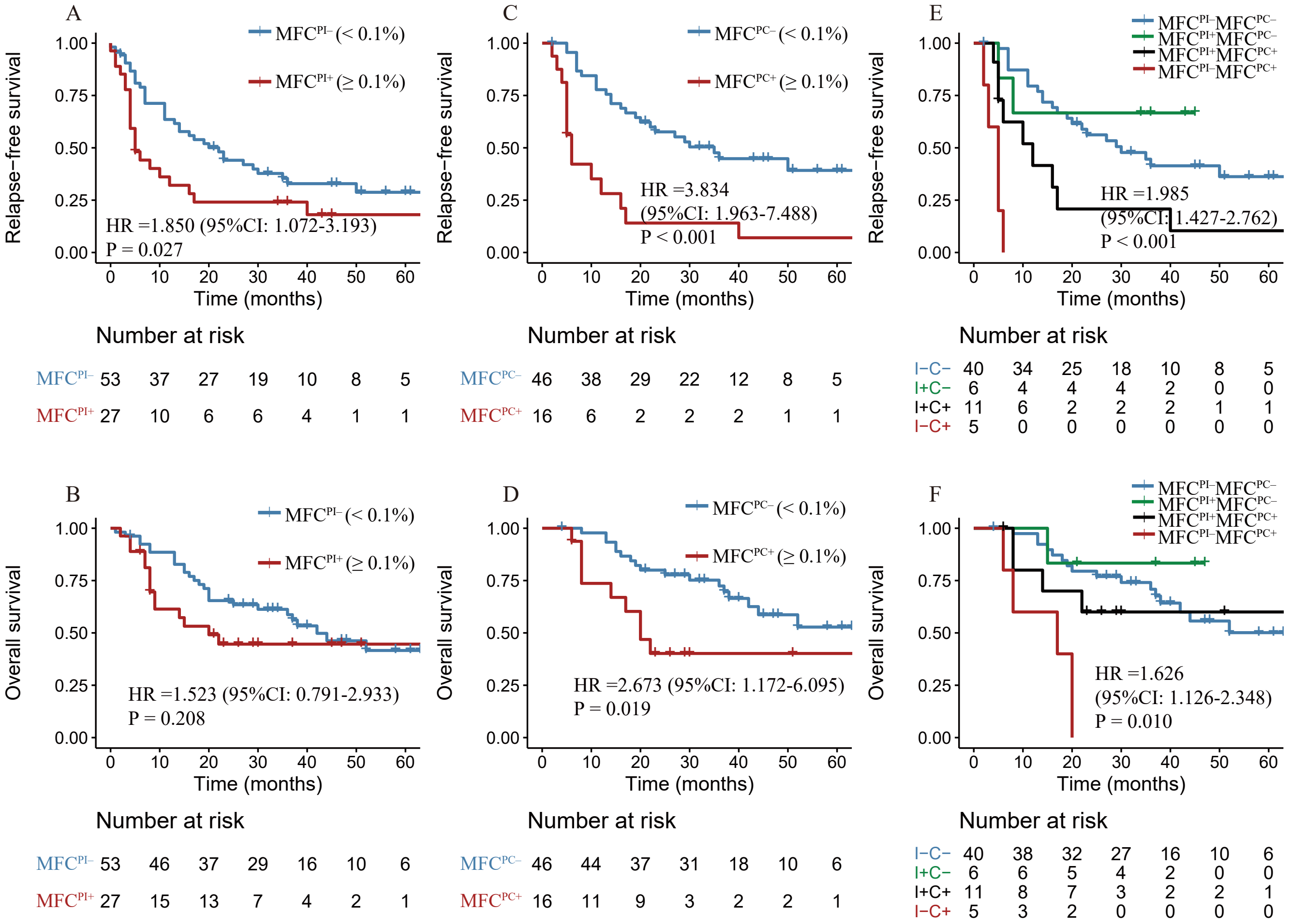
A
Relapse−free survival
MFC^{PI-} (< 0.1%)
MFC^{PI+} (≥ 0.1%)
HR =1.850 (95%CI: 1.072-3.193)
P = 0.027
Time (months)
Number at risk
MFC^{PI-} 53 37 27 19 10 8 5
MFC^{PI+} 27 10 6 6 4 1 1
C
Relapse−free survival
MFC^{PC-} (< 0.1%)
MFC^{PC+} (≥ 0.1%)
HR =3.834
(95%CI: 1.963-7.488)
P < 0.001
Time (months)
Number at risk
MFC^{PC-} 46 38 29 22 12 8 5
MFC^{PC+} 16 6 2 2 2 1 1
E
Relapse−free survival
MFC^{PI-}MFC^{PC-}
MFC^{PI+}MFC^{PC-}
MFC^{PI+}MFC^{PC+}
MFC^{PI-}MFC^{PC+}
HR =1.985
(95%CI: 1.427-2.762)
P < 0.001
Time (months)
Number at risk
I−C− 40 34 25 18 10 8 5
I+C− 6 4 4 4 2 0 0
I+C+ 11 6 2 2 2 1 1
I−C+ 5 0 0 0 0 0 0
B
Overall survival
MFC^{PI-} (< 0.1%)
MFC^{PI+} (≥ 0.1%)
HR =1.523 (95%CI: 0.791-2.933)
P = 0.208
Time (months)
Number at risk
MFC^{PI-} 53 46 37 29 16 10 6
MFC^{PI+} 27 15 13 7 4 2 1
D
Overall survival
MFC^{PC-} (< 0.1%)
MFC^{PC+} (≥ 0.1%)
HR =2.673 (95%CI: 1.172-6.095)
P = 0.019
Time (months)
Number at risk
MFC^{PC-} 46 44 37 31 18 10 6
MFC^{PC+} 16 11 9 3 2 2 1
F
Overall survival
MFC^{PI-}MFC^{PC-}
MFC^{PI+}MFC^{PC-}
MFC^{PI+}MFC^{PC+}
MFC^{PI-}MFC^{PC+}
HR =1.626
(95%CI: 1.126-2.348)
P = 0.010
Time (months)
Number at risk
I−C− 40 38 32 27 16 10 6
I+C− 6 6 5 4 2 0 0
I+C+ 11 8 7 3 2 2 1
I−C+ 5 3 2 0 0 0 0

Fig. S5

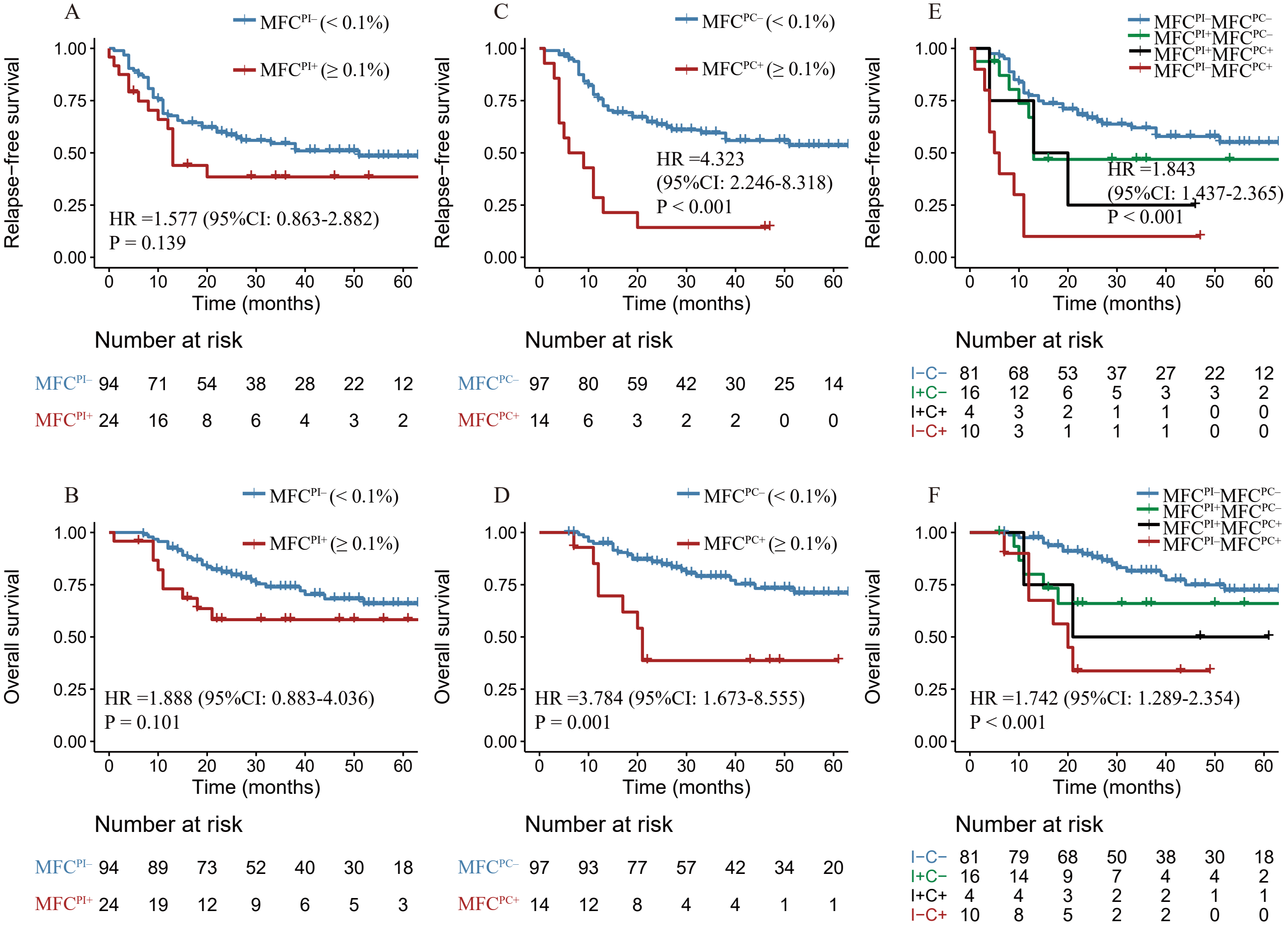
A
MFCPI– (< 0.1%)
MFCPI+ (≥ 0.1%)
Relapse−free survival
HR =1.577 (95%CI: 0.863-2.882)
P = 0.139
Time (months)
Number at risk
MFCPI– 94 71 54 38 28 22 12
MFCPI+ 24 16 8 6 4 3 2
C
MFCPC– (< 0.1%)
MFCPC+ (≥ 0.1%)
Relapse−free survival
HR =4.323
(95%CI: 2.246-8.318)
P < 0.001
Time (months)
Number at risk
MFCPC– 97 80 59 42 30 25 14
MFCPC+ 14 6 3 2 2 0 0
E
MFCPI–MFCPC–
MFCPI+MFCPC–
MFCPI+MFCPC+
MFCPI–MFCPC+
Relapse−free survival
HR =1.843
(95%CI: 1.437-2.365)
P < 0.001
Time (months)
Number at risk
I−C− 81 68 53 37 27 22 12
I+C− 16 12 6 5 3 3 2
I+C+ 4 3 2 1 1 0 0
I−C+ 10 3 1 1 1 0 0
B
MFCPI– (< 0.1%)
MFCPI+ (≥ 0.1%)
Overall survival
HR =1.888 (95%CI: 0.883-4.036)
P = 0.101
Time (months)
Number at risk
MFCPI– 94 89 73 52 40 30 18
MFCPI+ 24 19 12 9 6 5 3
D
MFCPC– (< 0.1%)
MFCPC+ (≥ 0.1%)
Overall survival
HR =3.784 (95%CI: 1.673-8.555)
P = 0.001
Time (months)
Number at risk
MFCPC– 97 93 77 57 42 34 20
MFCPC+ 14 12 8 4 4 1 1
F
MFCPI–MFCPC–
MFCPI+MFCPC–
MFCPI+MFCPC+
MFCPI–MFCPC+
Overall survival
HR =1.742 (95%CI: 1.289-2.354)
P < 0.001
Time (months)
Number at risk
I−C− 81 79 68 50 38 30 18
I+C− 16 14 9 7 4 4 2
I+C+ 4 4 3 2 2 1 1
I−C+ 10 8 5 2 2 0 0

Fig. S6

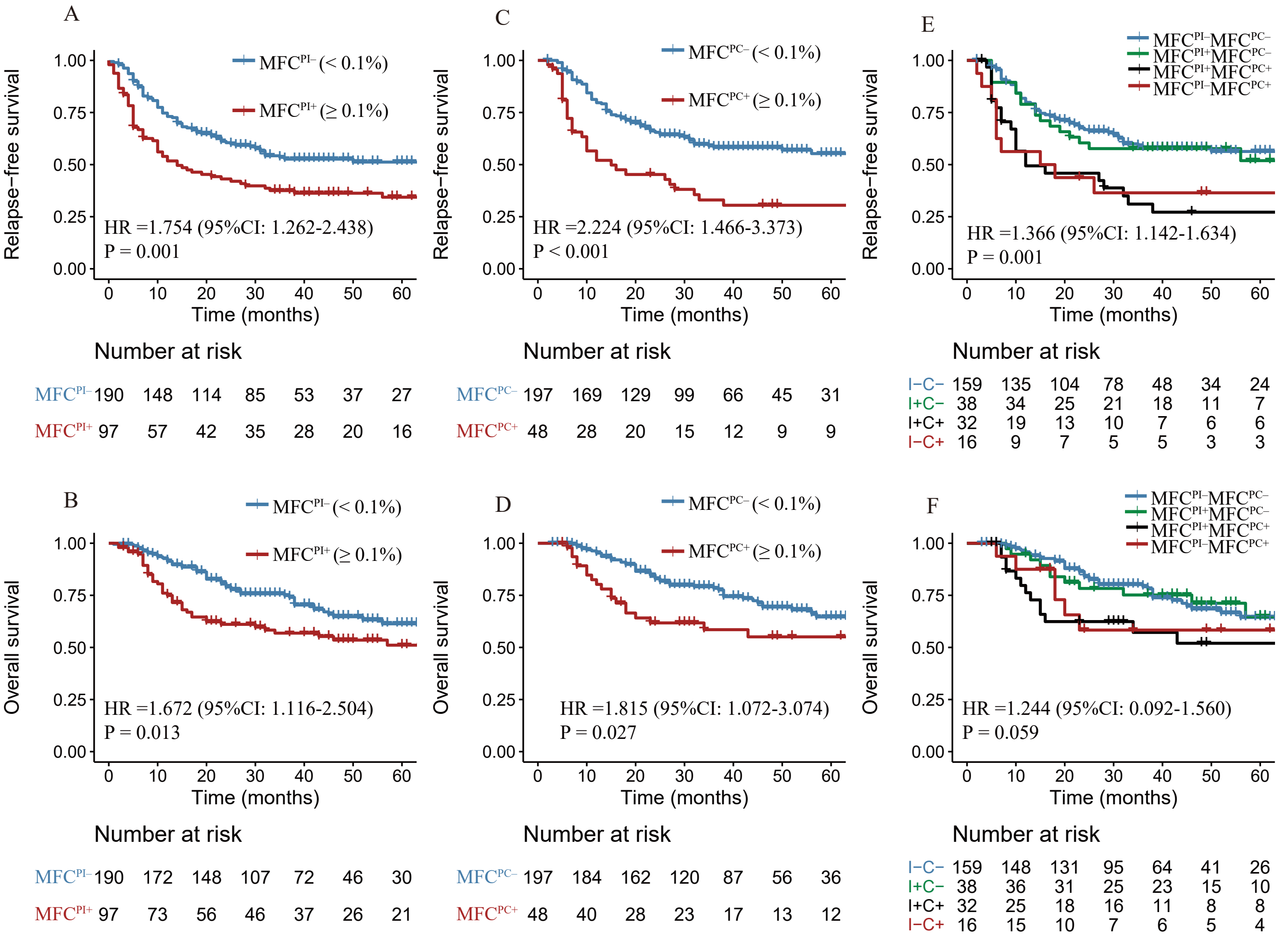
A
MFC^PI− (< 0.1%)
MFC^PI+ (≥ 0.1%)
Relapse−free survival
HR =1.754 (95%CI: 1.262-2.438)
P = 0.001
Time (months)
Number at risk
MFC^PI− 190 148 114 85 53 37 27
MFC^PI+ 97 57 42 35 28 20 16
C
MFC^PC− (< 0.1%)
MFC^PC+ (≥ 0.1%)
Relapse−free survival
HR =2.224 (95%CI: 1.466-3.373)
P < 0.001
Time (months)
Number at risk
MFC^PC− 197 169 129 99 66 45 31
MFC^PC+ 48 28 20 15 12 9 9
E
MFC^PI−MFC^PC−
MFC^PI+MFC^PC−
MFC^PI+MFC^PC+
MFC^PI−MFC^PC+
Relapse−free survival
HR =1.366 (95%CI: 1.142-1.634)
P = 0.001
Time (months)
Number at risk
I−C− 159 135 104 78 48 34 24
I+C− 38 34 25 21 18 11 7
I+C+ 32 19 13 10 7 6 6
I−C+ 16 9 7 5 5 3 3
B
MFC^PI− (< 0.1%)
MFC^PI+ (≥ 0.1%)
Overall survival
HR =1.672 (95%CI: 1.116-2.504)
P = 0.013
Time (months)
Number at risk
MFC^PI− 190 172 148 107 72 46 30
MFC^PI+ 97 73 56 46 37 26 21
D
MFC^PC− (< 0.1%)
MFC^PC+ (≥ 0.1%)
Overall survival
HR =1.815 (95%CI: 1.072-3.074)
P = 0.027
Time (months)
Number at risk
MFC^PC− 197 184 162 120 87 56 36
MFC^PC+ 48 40 28 23 17 13 12
F
MFC^PI−MFC^PC−
MFC^PI+MFC^PC−
MFC^PI+MFC^PC+
MFC^PI−MFC^PC+
Overall survival
HR =1.244 (95%CI: 0.092-1.560)
P = 0.059
Time (months)
Number at risk
I−C− 159 148 131 95 64 41 26
I+C− 38 36 31 25 23 15 10
I+C+ 32 25 18 16 11 8 8
I−C+ 16 15 10 7 6 5 4

Fig. S7

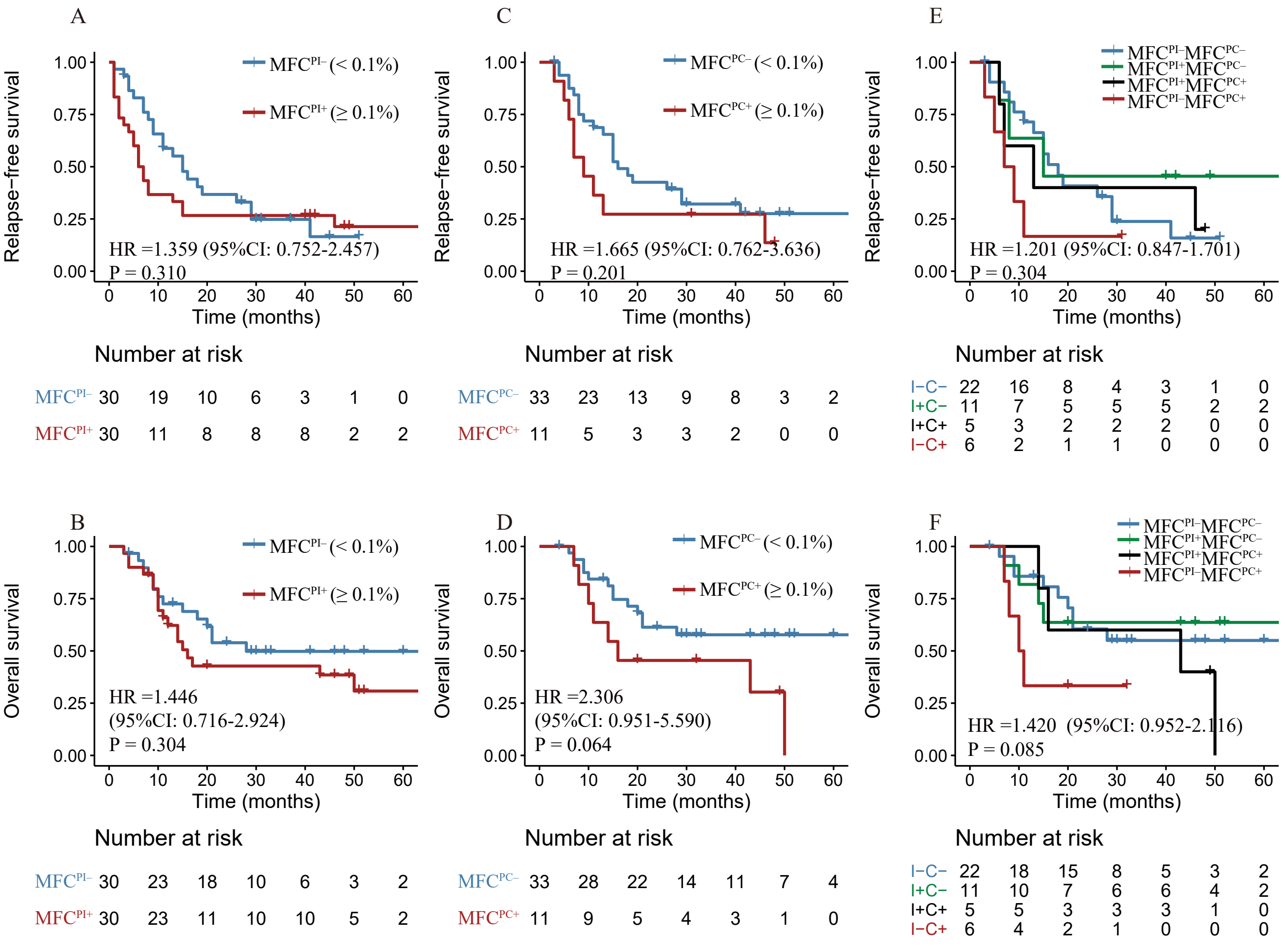
A
MFCPI− (< 0.1%)
MFCPI+ (≥ 0.1%)
Relapse−free survival
HR =1.359 (95%CI: 0.752-2.457)
P = 0.310
Time (months)
Number at risk
MFCPI− 30 19 10 6 3 1 0
MFCPI+ 30 11 8 8 8 2 2
C
MFCPC− (< 0.1%)
MFCPC+ (≥ 0.1%)
Relapse−free survival
HR =1.665 (95%CI: 0.762-3.636)
P = 0.201
Time (months)
Number at risk
MFCPC− 33 23 13 9 8 3 2
MFCPC+ 11 5 3 3 2 0 0
E
MFCPI−MFCPC−
MFCPI+MFCPC−
MFCPI+MFCPC+
MFCPI−MFCPC+
Relapse−free survival
HR =1.201 (95%CI: 0.847-1.701)
P = 0.304
Time (months)
Number at risk
I−C− 22 16 8 4 3 1 0
I+C− 11 7 5 5 5 2 2
I+C+ 5 3 2 2 2 0 0
I−C+ 6 2 1 1 0 0 0
B
MFCPI− (< 0.1%)
MFCPI+ (≥ 0.1%)
Overall survival
HR =1.446
(95%CI: 0.716-2.924)
P = 0.304
Time (months)
Number at risk
MFCPI− 30 23 18 10 6 3 2
MFCPI+ 30 23 11 10 10 5 2
D
MFCPC− (< 0.1%)
MFCPC+ (≥ 0.1%)
Overall survival
HR =2.306
(95%CI: 0.951-5.590)
P = 0.064
Time (months)
Number at risk
MFCPC− 33 28 22 14 11 7 4
MFCPC+ 11 9 5 4 3 1 0
F
MFCPI−MFCPC−
MFCPI+MFCPC−
MFCPI+MFCPC+
MFCPI−MFCPC+
Overall survival
HR =1.420 (95%CI: 0.952-2.116)
P = 0.085
Time (months)
Number at risk
I−C− 22 18 15 8 5 3 2
I+C− 11 10 7 6 6 4 2
I+C+ 5 5 3 3 3 1 0
I−C+ 6 4 2 1 0 0 0

Fig. S8

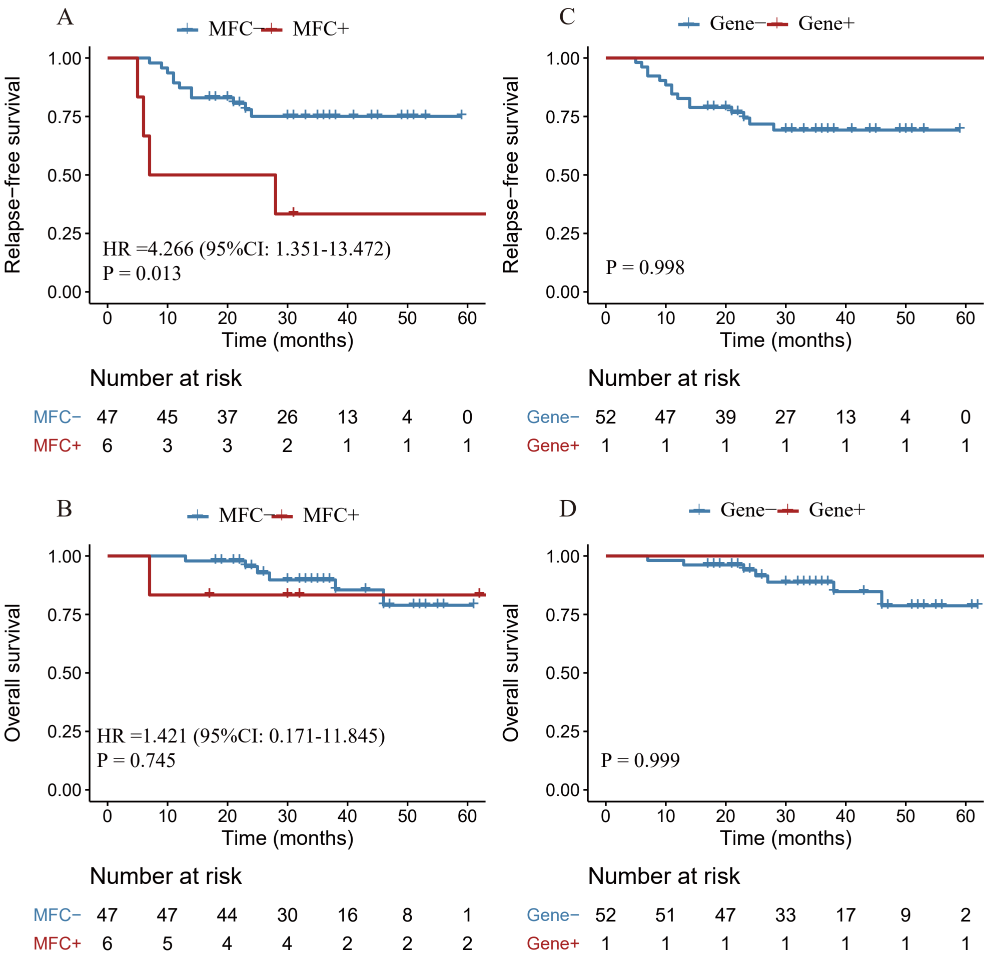
A
MFC− MFC+
Relapse−free survival
1.00
0.75
0.50
0.25
0.00
HR =4.266 (95%CI: 1.351-13.472)
P = 0.013
0 10 20 30 40 50 60
Time (months)
Number at risk
MFC− 47 45 37 26 13 4 0
MFC+ 6 3 3 2 1 1 1
C
Gene− Gene+
Relapse−free survival
P = 0.998
Time (months)
Number at risk
Gene− 52 47 39 27 13 4 0
Gene+ 1 1 1 1 1 1 1
B
MFC− MFC+
Overall survival
HR =1.421 (95%CI: 0.171-11.845)
P = 0.745
Time (months)
Number at risk
MFC− 47 47 44 30 16 8 1
MFC+ 6 5 4 4 2 2 2
D
Gene− Gene+
Overall survival
P = 0.999
Time (months)
Number at risk
Gene− 52 51 47 33 17 9 2
Gene+ 1 1 1 1 1 1 1

Fig. S9

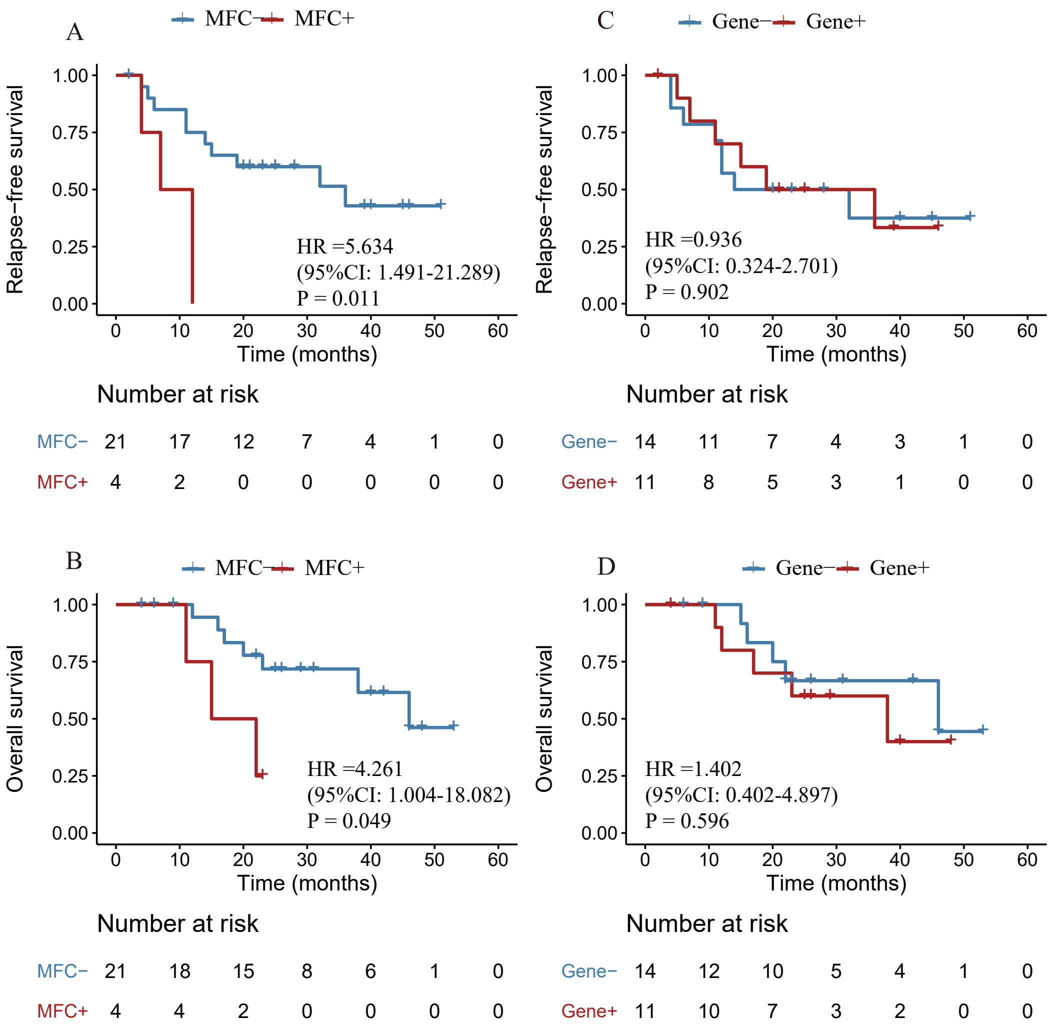
A
MFC− MFC+
Relapse−free survival
1.00
0.75
0.50
0.25
0.00
0 10 20 30 40 50 60
Time (months)
HR =5.634
(95%CI: 1.491-21.289)
P = 0.011
Number at risk
MFC− 21 17 12 7 4 1 0
MFC+ 4 2 0 0 0 0 0
C
Gene− Gene+
Relapse−free survival
HR =0.936
(95%CI: 0.324-2.701)
P = 0.902
Number at risk
Gene− 14 11 7 4 3 1 0
Gene+ 11 8 5 3 1 0 0
B
MFC− MFC+
Overall survival
HR =4.261
(95%CI: 1.004-18.082)
P = 0.049
Number at risk
MFC− 21 18 15 8 6 1 0
MFC+ 4 4 2 0 0 0 0
D
Gene− Gene+
Overall survival
HR =1.402
(95%CI: 0.402-4.897)
P = 0.596
Number at risk
Gene− 14 12 10 5 4 1 0
Gene+ 11 10 7 3 2 0 0
